# Prefix Imputation of Orphan Events in Event Stream Processing

**DOI:** 10.3389/fdata.2021.705243

**Published:** 2021-10-06

**Authors:** Rashid Zaman, Marwan Hassani, Boudewijn F. Van Dongen

**Affiliations:** Process Analytics Group, Faculty of Mathematics and Computer Science, Eindhoven University of Technology, Eindhoven, Netherlands

**Keywords:** event stream processing, online process mining, online conformance checking, prefix imputation, prefix-alignments

## Abstract

In the context of process mining, event logs consist of process instances called cases. Conformance checking is a process mining task that inspects whether a log file is conformant with an existing process model. This inspection is additionally quantifying the conformance in an explainable manner. Online conformance checking processes streaming event logs by having precise insights into the running cases and timely mitigating non-conformance, if any. State-of-the-art online conformance checking approaches bound the memory by either delimiting storage of the events per case or limiting the number of cases to a specific window width. The former technique still requires unbounded memory as the number of cases to store is unlimited, while the latter technique forgets running, not yet concluded, cases to conform to the limited window width. Consequently, the processing system may later encounter events that represent some intermediate activity as per the process model and for which the relevant case has been forgotten, to be referred to as orphan events. The naïve approach to cope with an orphan event is to either neglect its relevant case for conformance checking or treat it as an altogether new case. However, this might result in misleading process insights, for instance, overestimated non-conformance. In order to bound memory yet effectively incorporate the orphan events into processing, we propose an imputation of missing-prefix approach for such orphan events. Our approach utilizes the existing process model for imputing the missing prefix. Furthermore, we leverage the case storage management to increase the accuracy of the prefix prediction. We propose a systematic forgetting mechanism that distinguishes and forgets the cases that can be reliably regenerated as prefix upon receipt of their future orphan event. We evaluate the efficacy of our proposed approach through multiple experiments with synthetic and three real event logs while simulating a streaming setting. Our approach achieves considerably higher realistic conformance statistics than the state of the art while requiring the same storage.

## 1 Introduction

Process mining discipline bridges the gap between data science and process science (Van Der Aalst, 2016). Taking event data as input, its various techniques provide insights into the underlying business process. An event, for process mining tasks, refers to the execution of activities as part of a process instance, while process instances are referred to as cases. Process mining based on historical event data, also referred to as post-mortem analysis, operates on static past event data consisting usually of completed cases. In contrast, online process mining techniques are tailored to process event streams for continuously discovering insights into running cases. Online conformance checking techniques gauge the harmony between running cases of the streaming event logs and the existing process model, also referred to as the reference process model.

Event streams, being the input of online conformance checking techniques, inherit the key characteristics of the data streams (Golab and Özsu, 2003; Aggarwal, 2007; Bifet et al., 2010). The source of an event stream is a business process where the process activities exhibit some behavioral relation, for instance, causality. The events generated as a result of the execution of these business process activities accordingly embody the source relation. Online conformance checking techniques, rather than individual events, look at a group of events related under the notion of a case. This behavioral relation between events belonging to the same process instance consequently results in added complexity for dealing with event streams.

**FIGURE 1:**
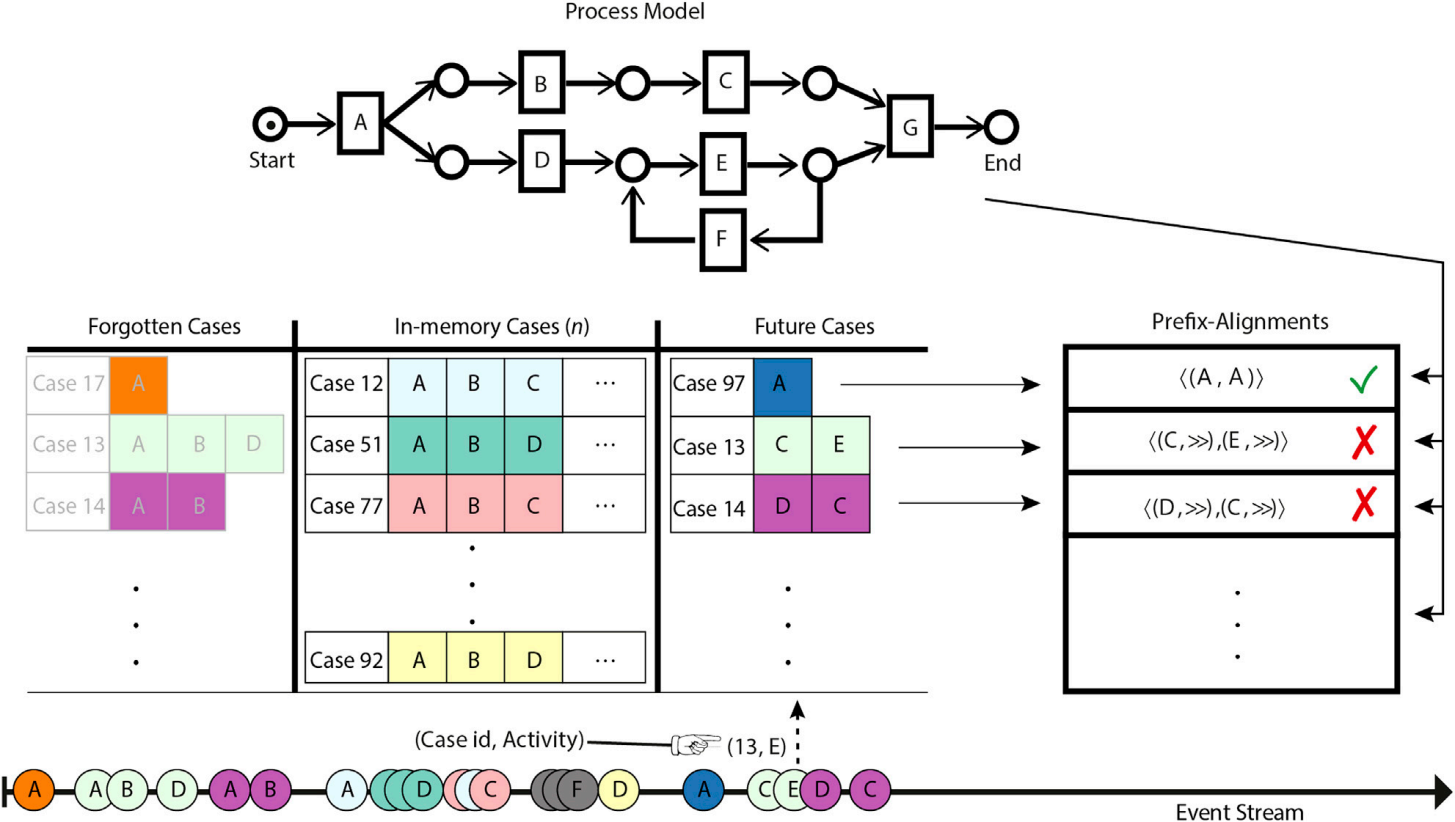
Schematic overview of the bounded-memory online conformance checking.

Referring to Figure 1, multiple event sources emit events at a very high rate on the event stream. A stream event minimally contains the relevant case id and the name of the activity executed in the context of this case. The events on the stream are observed by the stream conformance checking system. For the sake of illustration, we have uniquely color-coded the events belonging to the same case. Observed events are stored in the memory of the system by appending them to the prefixes of their relevant cases already existing in the memory. If the prefix for a relevant case of an observed event does not exist in the memory, then a new entry for the relevant case is created implying that a fresh case has been initiated. In either situation, the case is then subjected to a comparison with the reference process model, by the prefix-alignment–based conformance checking technique, to highlight deviations, if any. The prefix-alignments of the cases keep evolving with the evolution of the cases, i.e., through observing their future events.

Burattin and Carmona (2017) summarized the key assumptions from literature for dealing with streams (Hassani, 2015). Two of these assumptions are related to the bounded nature of the available memory and stated as *“algorithms processing data streams should be able to process an infinite amount of data, without exceeding memory limits”* and *“the amount of memory available to an algorithm is considered finite and typically much smaller than the data observed in a reasonable span of time.”* However, the majority of the state-of-the-art online process mining approaches do not take into consideration these assumptions in spirit.

State-of-the-art online conformance checking techniques bound memory through delimiting either the number of events or the number of cases to be simultaneously retained in memory. In other words, these techniques keep forgetting events or cases in overflow situations. Delimiting the number of events to be retained per case still requires an unlimited number of cases to be stored. Limiting the number of cases, as adapted by Burattin et al. (2018), forgets cases on the basis of inactivity. The cases that have not been updated for long are considered to be inactive and therefore deemed the most suitable candidates to be forgotten. Referring to Figure 1, the system has *n* bounded memory, i.e., a maximum of *n* number of cases can be stored simultaneously. In an overflow situation, i.e., the number of observed cases surpassing the memory limit, cases are forgotten out of the memory on the criteria of inactivity.

The inactivity-based forgetting criteria may not always be optimal as, due to a variety of contextual factors, different cases exhibit a different temporal distribution of events. The forgotten cases may not necessarily be completed or permanently halted, and events belonging to these cases may pop up in the future. We refer to such events as orphan events and their respective cases as prefix-missing cases. Afterwards, orphan events corresponding to the forgotten cases are observed on the stream. Processing such prefix-missing cases as they are may result in elicitation of unreliable insights into the business process. As depicted in Figure 1, in the future, along with fresh cases, orphan events for the cases forgotten because of the memory constraint are observed on the stream. Upon subjection to prefix-alignment–based online conformance checking, the orphan events, having lost their prefix, are declared as non-conforming to the reference process model.

A simple approach to deal with prefix-missing cases would be to adapt the listwise deletion mechanism by neglecting them for conformance checking. This approach is problematic for at least two reasons. First, as proposed by van Zelst et al. (2019), we need to keep track of the forgotten cases so that we may ignore any future residual events belonging to these cases. This record-keeping may itself grow unbounded with the passage of time, or stream to be precise. Second, in some process execution environments, the arrival rate of new process instances may be high such that, at any point in time, the number of process instances running in parallel surpasses the case bounds imposed on the memory. The proposed listwise deletion in this scenario will affect a considerable portion of the observed cases. Particular variants of cases, such as those having a sparse distribution of events, may always be forgotten and accordingly not be taken into consideration.

To keep the memory bounded but at the same time counteract its missing-prefix effect, we propose a novel two-step solution in this work. In the *forgetting* step, we tactfully forget the cases in accordance with set criteria, when required. In the *imputation* step, through positioning in a normative process model, the observed orphan events are imputed with a suitable prefix. The forgetting mechanism provides leverage for the imputation step by selectively forgetting cases that can be reliably reproduced. Through this inter-twined approach, we ensure on the one hand to bound the memory and on the other hand to enable the orphaned cases to be properly handled in conformance checking.

To evaluate the efficacy and viability of our approach, we conduct extensive experimental evaluations with both synthetic and real event data. The results are promising. In our experimental setup, we have placed the proposed approach as overlay on the prefix-alignment–based online conformance checking technique of van Zelst et al. (2019). Nevertheless, the approach is generic and can be tailored to any online process mining technique.

To summarize, the contributions of this work are 1) devising a systematic forgetting mechanism to bound memory in event stream processing systems and 2) developing a process-model–based prefix-imputation technique to treat prefix-missing cases by leveraging the forgetting mechanism for this purpose.

The rest of this paper is organized as follows. In Section 2, we present a broad overview of the related work. Section 3 provides the definitions and necessary details of the concepts fundamental to our proposed approach. Section 4 presents the key idea and components of our proposed approach. Section 5 discusses the implementation, experiments, results, and key findings of the experiments. Section 6 concludes this paper along with some reflection on the opportunities for future work.

## 2 Related Work

With process mining matured as a discipline, online process mining is its emerging branch. Burattin et al. (2014) and Hassani et al. (2015) are the two pioneering works in the field of online process discovery. Hassani et al. (2019) explored the potential application of sequential pattern mining in online process discovery. In contrast to online process discovery, the online conformance checking has received sufficient attention from the research community. Burattin and Carmona (2017) and Burattin (2017), using regions theory, extended the transition system of the reference process model with potential deviations and associated them with costs larger than zero. A transition system is a basic process modeling notation, consisting of states and transitions (Van Der Aalst, 2016). Traces, the sequences of recorded events for cases, are replayed on the extended transition system, and those accumulating non-zero costs are considered non-conformant.

Alignments (Adriansyah, 2014) are considered the *de facto* standard underlying technique for conformance checking of traces. Prefix-alignments, a variant of conventional alignments able to deal with incomplete cases, have been adapted by van Zelst et al. (2019) for conformance checking in streaming environments. Burattin et al. (2018) proposed a less compute-intensive conformance checking approach, as compared to alignments, for streaming event environments. Their proposed approach gauges the non-conformance of cases through comparing the behavioral patterns of the observed events with the patterns constituting the reference process model.

Dealing with unbounded evolving data streams through bounded memory has been one of the major challenges. The problem is more aggravated in the case of event streams as the data points, i.e., events, are behaviorally connected. Hassani et al. (2019), similar to the techniques prevalent in data stream processing such as *summarization* (Bahri et al., 2021; Gomes et al., 2019), suggested maintaining an abstract intermediate representation of the stream to be used as input for various process discovery techniques. Online conformance checking techniques in general assume unbounded memory. Burattin et al. (2018) limited the number of in-memory cases by forgetting *inactive* cases. Although their behavioral-pattern–based conformance checking mechanism can deal with incomplete cases, the reported conformance statistics are no more global optimum. Furthermore, the case-related *completeness* and *confidence* scores, complementary to the conformance statistics, are over- or underestimated. Forgetting traces on vague criteria such as inactivity leads to the missing-prefix problem. A variant of van Zelst et al. (2019) bounds the number of events (or moves in terms of alignments) per trace to be revisited during the appropriation of their previously calculated alignments, but the number of traces to store is still boundless.

Event data quality issues have been adequately researched in the perspective of historic data with supposedly completed cases. Jagadeesh Chandra Bose et al. (2013) made the first effort to exhaustively list the potential event data problems. Wang et al. (2013) presented a pioneering work for treating noisy event data and proposed a backtracking algorithm for the recovery of a case. Andrews et al. (2018) envisioned an event log query language that directly detects log imperfection issues. Suriadi et al. (2017) adapted a pattern-based approach to identify and repair event log quality issues. Sim et al. (2019) proposed an *event chain*–based solution to repair missing activities as well as missing resources and attributes. Kong et al. (2019) predicted missing events through identifying *sound conditions* in the log. Dixit et al. (2018) proposed a solution for repairing ordering-related imperfections in event logs. Almost all of these mentioned works are model-agnostic.

Rogge-Solti et al. (2013) presented a model-based approach that combines stochastic Petri nets, alignments, and Bayesian networks to predict missing events and their timestamps. Cheng and Kumar (2015) proposed a technique to sanitize noisy logs by first building a classifier on a subset of the log and then applying the classifier rules to remove noisy traces from the log. The above-mentioned noise-cleaning techniques mostly treat single event noise, with access to intra-case and inter-case events. In contrast, our prefix-imputation approach is aimed at streaming environments and making imputation decisions on the basis of a single orphan event.

Regarding event data quality issues in stream processing, van Zelst et al. (2020) built a probabilistic automaton to model a time-evolving subset of the behavior of the total event stream to filter out spurious events. In a memory-bounded stream setting, the mentioned approach will most probably treat the orphan events as spurious and, in line with pairwise deletion, filter them out. In contrast, our proposed approach restores the utility of orphan events. Awad et al. (2020) used window and speculative-based techniques for discovering reliable directly-follows graphs even with events arriving out-of-order. To the best of our knowledge, missing prefixes have been investigated as an event data quality issue neither in historic data nor in the context of event streams. This is the first work that actually addresses memory boundedness in event stream processing through a holistic approach by systematically forgetting cases and accordingly imputing the prefix-missing cases with a suitable prefix, on the basis of the reference process model.

## 3 Preliminaries

In this section, we provide the definitions and necessary details of some concepts which are fundamental to our proposed approach.

### 3.1 Process Model

Business process models are one of the first-class citizens in process mining analysis and are required as input in almost all sorts of process mining techniques. A business process model formally represents a real-life process. Business *process models* as Petri nets are represented with a tuple *N* = (*P*, *T*, *F*, *λ*), where *P* represents a finite set of places, *T* is a finite set of transitions, and *F* ⊂ (*P* × *T*) ∪ (*T* × *P*) is a set of flow relations between places and transitions. *λ* is a labeling function assigning transitions *T* with labels from the set of the activity labels Λ.

The state of a process instance is represented through a *marking* *M*, as a multiset of tokens over the places *P* in the process model *N*, i.e., M:P→N . Furthermore, *M*_*i*_ is an initial marking and *M*_*f*_ is a final marking. Elements of *P* ∪ *T* are called *nodes*. For a node *y* ∈ *P* ∪ *T*, a node *x* is said to be its input node if there is a direct arc *f* ∈ *F* from *x* to *y*. A node *z* is said to be the output node of *y* if there is a direct arc *f* ∈ *F* from *y* to *z*. •*y* represents the set of all input nodes of *y*, while *y*• represents the set of all of its output nodes. Silent transitions *τ* (taus) are essentially used for the completion of routing in Petri nets.

**FIGURE 2:**
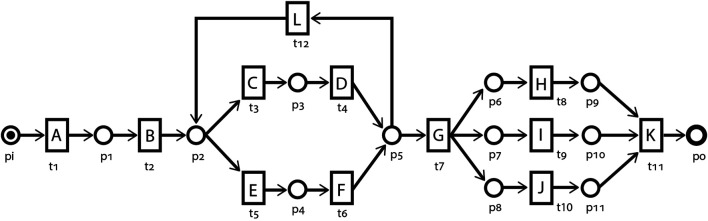
An example of a process model represented as a Petri net *N*, consisting of places {*p*_1_, *p*_2_, … } and transitions {*t*_1_, *t*_2_, … } labeled with activity names {*A*, *B*, … } and inter-connected through directed arcs *F*.

Figure 2 depicts an example process model as a Petri net. The circles {*p*_1_, *p*_2_, … } represent places. Rectangle-shaped transitions {*t*_1_, *t*_2_, … } map the process model to the corresponding process activities through labels {*A*, *B*, *C*, … }. Places and transitions are connected through directed arcs *F*, conventionally without labels. Behaviorally, transitions *t*_1_ and *t*_2_ are in a *sequential* relation and, in some processes, may be in a *causal* relation. Transitions {*t*_3_, *t*_4_} are in a sequential relation but in a *choice* relation with transitions {*t*_5_, *t*_6_}. Transition *t*_12_ enables *looping*, i.e., multiple runs, of {*t*_3_, *t*_4_} and {*t*_5_, *t*_6_}. Transitions {*t*_8_, *t*_9_, *t*_10_} are in a *parallel* or *concurrency* relation. The output arcs of transition *t*_7_ instantiate an *AND-split* pattern which enables concurrency, while the *AND-join* pattern constituted by the input arcs of *t*_11_ synchronizes the concurrent behavior. With the start place *p*_*i*_ having a single token, the Petri net is in the initial marking $\left[{p_{i}}^{1}\right]$ , or simply [*p*_*i*_].

A marking *M* can *simply* be represented in terms of the input or output places of the transitions. A transition *t* having a token in each of its input places •*t* is said to be enabled, i.e., •*t* ≤ *M*. An enabled transition can fire, thereby consuming a token from each of its input places •*t* and accordingly producing a token in each of its output places *t*•, resulting in a new marking *M*′ = *M* −• *t* + *t*•. This firing is denoted *M*[ *t*⟩*M*′. In this work, we refer to the set of transitions enabled in the initial marking *M*_*i*_ as *case starters* *T*_*ini*_. The consecutive firing of a set of (enabled) transitions starting from a marking *M* is referred to as a *firing or execution sequence* of *M*. We will be using the two terms interchangeably in this work. A marking *M* is *reachable* from the initial marking *M*_*i*_ if and only if there exists a sequence of enabled transitions whose firing leads *M*_*i*_ to *M*. Typically, the set of firing sequences $P_{N}$ for a process model *N* is finitely large in the absence of loops and infinitely large in the presence of a loop. A firing sequence starting from *M*_*i*_ and ending in *M*_*f*_ will be referred to as a *complete firing sequence* of *N*.

We define the *transition prefixes* for a transition *t* ∈ *T* as the set of prefixes $P_{t}\subset P_{N}$ consisting of the firing sequences corresponding to all the reachable markings *M* of *M*_*i*_ which includes at least •*t*, i.e., •*t* ≤ *M*. As with $P_{N}$ , $P_{t}$ can be infinitely large in the presence of loops preceding the transition *t*. The length of a prefix $pref\in P_{t}$ , i.e., the number of transitions in the firing sequence, is denoted |*pref*|. Furthermore, a prefix $pref_{t}\in P_{t}$ is a *shortest prefix* for transition *t* if for all $pref\in P_{t}$ it holds that |*pref*| ≥ |*pref*_*t*_|. We introduce the notion of *non-deterministic transition* in this work. A transition is considered *non-deterministic* if at least one of its prefixes $pref\in P_{t}$ leads to a marking *M* such that *M* − •*t* ≠ *∅*.

Referring to Figure 2, in the initial marking *M*_*i*_ of the process model, only transition *t*_1_ is enabled and therefore is the only *case starter*, i.e., *T*_*ini*_ = {*t*_1_}. Firing *t*_1_ results in the marking [*p*_1_]. ⟨*t*_1_, *t*_2_, *t*_3_⟩ is one of the infinite sets of firing sequences of this process model, resulting in the marking [*p*_3_]. The marking [*p*_3_] is therefore a reachable marking of the initial marking *M*_*i*_. The firing sequence ⟨*t*_1_, *t*_2_, *t*_3_, *t*_4_, *t*_12_, *t*_5_, *t*_6_, *t*_7_, *t*_8_, *t*_9_, *t*_10_, *t*_11_⟩ represents one of the possible complete firing sequences. The set of firing sequences {⟨*t*_1_, *t*_2_, *t*_3_, *t*_4_⟩, ⟨*t*_1_, *t*_2_, *t*_3_, *t*_4_, *t*_12_, *t*_3_, *t*_4_⟩, ⟨*t*_1_, *t*_2_, *t*_3_, *t*_4_, *t*_12_, *t*_5_, *t*_6_⟩, … } represents the transition prefixes for transition *t*_7_, with ⟨*t*_1_, *t*_2_, *t*_3_, *t*_4_⟩ being the shortest prefix. In the context of our proposed approach, transition *t*_8_ is an example of a non-deterministic transition as firing any of its transition prefixes results in a marking that contains tokens in places additional to *p*_6_. For instance, the shortest prefix ⟨*t*_1_, *t*_2_, *t*_3_, *t*_4_, *t*_7_⟩ results in a marking [*p*_6_, *p*_7_, *p*_8_].

Soundness, liveness, and boundedness are some important and desirable properties of a Petri net, which are out of the scope of this paper, and interested readers are referred to Van Der Aalst (2016). We will be mainly considering *workflow nets* in this paper which are Petri nets having a distinct source place *start* and a distinct sink place *end*, and all other nodes are on a path from *start* to *end*.

### 3.2 Events

Along with a process model, event logs and its related concepts such as cases, activities, and timestamps are also treated as first-class citizens in process mining analysis. The execution of activities in an organizational business process leaves a footprint in the information system of the organization in the form of *events*. Typically, this information is extracted from the information system as an *event log* under the notion of *cases*. A case refers to a particular process instance on the level of a process or system. For instance, in the business process of an insurance company, an individual insurance claim is referred to as a case.

An *event* refers to the atomic execution of a specific activity as part of a business process. An event *e* minimally consists of 1) the case or process instance identifier to which the event belongs, 2) the corresponding activity name represented as *#*_*activity*_(*e*), and 3) the timestamp of the execution of the corresponding activity represented as *#*_*time*_(*e*). It is important to note that every event is unique and distinct. Events referring to exactly the same activity, having the same timestamp, and belonging to the same case of a process are by context two different and distinct events.

The sequence of events *σ* corresponding to activities executed in the perspective of a particular case is treated collectively under the notion of a *trace*. |*σ*| denotes the length, i.e., the number of events, of a trace. Two event sequences can be concatenated, i.e., *σ*_1_ ⋅ *σ*_2_. Similarly, an event *e* can be concatenated to a sequence, i.e., *σ* ⋅ *e*. For the sake of simplicity, we represent traces as a sequence of the respective activities of the events. In some contexts, the terms *trace* and *case* are used interchangeably. For a trace *σ* of length *n*, we define its prefixes $P_{\sigma }$ as the set containing all of its length *k* heads where 0 ≤ *k* ≤ *n*. We call $P_{\sigma }$ the *trace prefixes* of *σ*. An *event log* *L* is a partially ordered list of events *e* belonging to a single or multiple cases of a business process. Event logs can also be *simply* represented as a multiset of traces. Each trace in this multiset is referred to as a *trace variant*. For simplicity, as with events, we represent event logs as a multiset of traces containing sequences of the respective activities of the events, i.e., *#*_*activity*_(*e*).

Table 1 depicts an excerpt of example log *L* obtained through the firing of some firing sequences of the process model of Figure 2. Each row in Table 1 represents an event *e*. For instance, the first row is an event *e*_1_ with the event id of “1” and corresponds to execution of the activity *#*_*activity*_(*e*) = *A* in the context of Case “7” and at timestamp *#*_*time*_(*e*) = *“*2021−01−17 12:45.” Events *e*_7_, *e*_8_, and *e*_9_ constitute a trace which corresponds to Case “21,” which in essence is a firing sequence of the process model depicted in Figure 2. The trace corresponding to Case “7” can simply be denoted in the sequence of activities as ⟨*A*, *B*, *C*, *G*⟩. The event log of Table 1 can be represented simply, i.e., a multiset of trace sequences, as *L* = [⟨*A*,*B*,*C*,*G*⟩^1^, ⟨*A*,*B*,*C*⟩^1^, ⟨*A*,*B*,*E*⟩^1^]. It is relevant to note that Case “7” and Case “13” are running in parallel. Streaming event environments are characterized by a large number of such in-parallel running cases.

**TABLE 1:** An example event log excerpt. Each row is an event and corresponds to the execution of an atomic activity in the process. An event shall minimally contain the values for a case identifier, an activity name, and a timestamp.

Event ID	Case ID	Activity	Timestamp
1	7	A	2021-01-17 12:45
2	13	A	2021-01-17 13:03
3	7	B	2021-01-18 10:07
4	13	B	2021-01-18 10:57
5	7	C	2021-02-03 14:31
6	13	C	2021-02-03 17:29
7	21	A	2021-03-19 16:49
8	21	B	2021-03-19 16:59
9	21	E	2021-03-20 11:23
10	7	G	2021-03-21 17:07
⋮	⋮	⋮	⋮

### 3.3 Event Streams

An event log is a static and scoped view of the process where further addition of cases or events is not possible. In contrast, an *event stream* is a dynamic and evolving view of the events, with already observed cases evolving and new cases being added to the stream. Formally, let *C* be the universe of case identifiers and A be the universe of possible activities. An event stream *S* is an infinite sequence of events over C×A , i.e., $S\in {\left(C\times A\right)}^{*}$ . A stream event is usually represented as (c,a)∈C×A , denoting that activity *a* has been executed in the context of case *c*. As with events in general, every stream event is unique and distinct, even if their respective activities, the case identifiers, and even the arrival times are exactly the same. Observed stream events are stored under the notion of their respective cases in a *case administration* *D*_*C*_. The case administration *D*_*C*_ can be bounded by the maximum number of cases it is allowed to store. We consider a stream event as an *orphan* *o* = (*c*, *a*) if a trace prefix for its case *c* does not exist in the case administration *D*_*C*_ and its activity *a* does not match the label of any of the transitions in *case starters*, i.e., *T*_*ini*_. Accordingly, the case *c* of the orphan event *o* will be referred to as a prefix-missing case.

Event streams are characterized by continuous and unbounded emission of stream events (*c*, *a*) by in-parallel running cases. Event stream processing systems are equipped with finite memory and limited processing capabilities. Additionally, their response time is required to be bounded. Due to memory limitations, such systems cannot store all previously observed events. Similarly, due to the constraints on response times and limited processing capabilities, the stored events are supposed to be accessed and processed in a single pass (Hassani, 2015; Hassani et al., 2019). Event stream processing systems lack any access or explicit knowledge about future events.

### 3.4 Prefix-Alignments

Alignments are considered the *de facto* standard technique for conformance checking of cases. Alignments explain the sequence of events (or simply activities) in a trace through a complete firing sequence of the reference process model (Carmona et al., 2018). A case is considered completely conformant or fitting if a complete firing sequence of the process model that fully explains its trace can be discovered. Otherwise, the trace is reported as non-conformant. The extent of the non-conformance of cases is then measured through the difference of their trace from a maximally explaining complete firing sequence.

**FIGURE 3:**
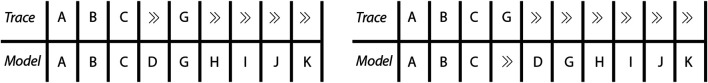
Two example alignments for Case “7” of event log of Table 1 with the process model of Figure 2. Both example alignments relate the trace of Case “7” with a complete firing sequence of the process model but differ in the number of synchronous, model, or log moves.

Consider the trace for Case “7,” i.e., ⟨*A*, *B*, *C*, *G*⟩, of the event log’s excerpt depicted in Table 1. Figure 3 shows two of the many possible alignments of this trace with the Petri net of Figure 2. The *trace* part of the alignment (neglecting ≫’s) is the same as the trace for Case “7,” while the *model* part of the alignment (neglecting ≫’s) is a complete firing sequence of the Petri net. A pair of the corresponding trace and model entry in the alignment is known as a *move*, for instance, *(A, A)*. The moves with no skip symbol ≫ in either part of the pair are termed *synchronous moves*. Synchronous moves imply that an enabled transition with the same label as the trace activity of the pair is available in the marking reachable by firing the transitions in the preceding pairs. In both alignments of Figure 3, the first three activities can be explained by transitions in a firing sequence of the process model.

Moves with ≫ in the trace part of the pair are referred to as *model moves* and illustrate that the trace is missing an activity for the transition enabled in the marking reachable by firing the transitions in the preceding pairs. For instance, the fourth move in the left alignment of Figure 3 is a model move as the transition with label *D* is enabled but an event with activity *D* is missing in the trace at the required position. Similarly, moves with ≫ in the model part of the pair are referred to as *log* or *activity moves*. Log moves signal the missing of an enabled transition in the process model for the trace activity of the pair. For instance, in the right alignment of Figure 3, after the first three synchronous moves, an enabled transition with label *G* cannot be found for the activity *G* of the trace.

As evident from Figure 3, multiple alignments are possible for a trace and model. Therefore, moves are associated with a *move cost* in order to manipulate and rank different alignments of a trace. Usually, synchronous moves and model moves with silent transitions (≫, *τ*) are assigned a zero cost. The sum of the costs of all the individual moves of an alignment is referred to as the *(raw) trace fitness cost*. Conformance checking looks for an optimal alignment *γ*_*opt*_ which bears the least trace fitness costs. In Figure 3, the left alignment is optimal with respect to the right alignment, assuming a cost of 1.0 for log, model moves and 0.0 for synchronous moves and model moves with silent transitions (≫, *τ*). It is worth mentioning that multiple optimal alignments may exist for the same trace.

Conventional alignments assume the cases to be completed such that no further events are to be observed for these cases. Therefore, alignments take into consideration complete firing sequences of the reference process model for explaining the traces of these cases. In event streams, the cases continuously evolve and the corresponding traces may essentially not represent a complete firing sequence of a Petri net, but rather a prefix. For checking the conformance of such evolving cases, the prefix-alignment variant of the conventional alignments is more appropriate. *Prefix-alignments* explain the sequence of events (or simply activities) in a trace through a firing sequence of the process model, rather than a complete firing sequence. The rest of the concepts, such as moves and their associated costs, are common with the conventional alignments.

**FIGURE 4:**
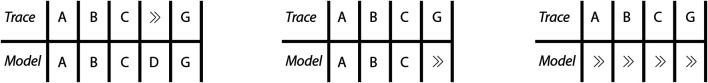
Three example prefix-alignments for Case “7” of event log of Table 1 with the process model of Figure 2. All these example prefix-alignments relate the trace of Case “7” with a partial firing sequence of the process model but differ in the number of synchronous, model, or log moves.

Consider the trace for Case “7,” i.e., ⟨*A*, *B*, *C*, *G*⟩, of the event log’s excerpt depicted in Table 1. Figure 4 shows three of the possible prefix-alignments of this trace with the Petri net of Figure 2. The *trace* part of the prefix-alignment (neglecting ≫’s) still corresponds to the trace, while the *model* part of the prefix-alignment (neglecting ≫’s) is a prefix of a complete firing sequence of the process model of Figure 2. As with conventional alignments, different types of moves in prefix-alignments are also associated with move costs in order to manipulate and rank prefix-alignments of a trace for identifying the optimal one. The optimal prefix-alignment evolves and changes with the evolution of the trace. Interested readers are referred to Carmona et al. (2018) for a deeper understanding of conformance checking, alignments, prefix-alignments, and related concepts.

## 4 Model-Based Prefix Imputation

In this section, we present the details of our proposed approach. We are scoping our approach to the case-based memory bounds where we have a fixed window of width *n*, i.e., we choose *n* to be the maximum number of cases allowed to be stored in *D*_*C*_ simultaneously. Additionally, we are considering the *inactivity*-based case forgetting in memory overflow situations as our *competitor approach*. The prefix-alignment approach of van Zelst et al. (2019) is used by default for online conformance checking, unless stated otherwise.

**FIGURE 5:**
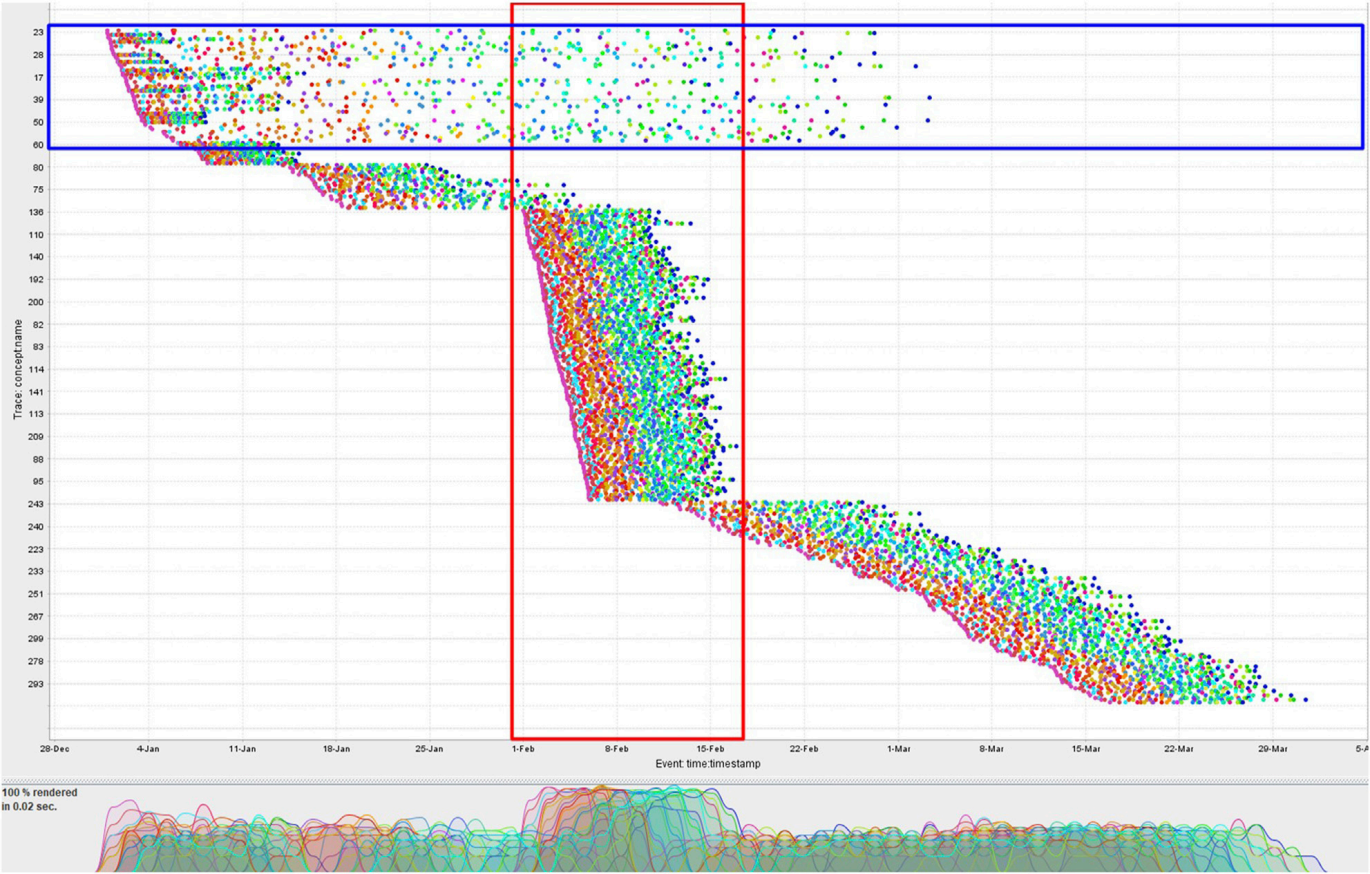
Dotted chart visualization of the CCC’19 event data. Cases are depicted with respect to their actual arrival time. Each point corresponds to an event, and each color corresponds to a distinct class of events. Events for cases are horizontally distributed.

Consider an event streaming environment where the number of in-parallel running cases can surpass the upper bound *n* of *D*_*C*_. The events arrive with varying distribution for different cases. The dotted chart visualization of CCC’19 event data (Munoz-Gama et al., 2019) in Figure 5 can be considered a snapshot of such a stream. Every dot in the chart represents an event, with each event type having a distinct color. This dotted chart illustrates the variation in the temporal distribution of the events among the cases. The majority of the cases covered by the red-colored box are running in parallel.

### 4.1 Missing-Prefix Problem

Consider the state of the event stream processing system where *n* distinct cases have already been observed and accordingly stored in *D*_*C*_. An event (*c*, *a*) belonging to a case other than the already stored *n* cases, i.e., *c* ∉ *D*_*C*_, is observed. Let us refer to this case as the (*n* + 1)th case. As per the competitor approach for forgetting cases, a (supposedly) inactive case in some intermediate marking *M* ≠ *M*_*f*_ is forgotten and memory is freed up for the recently observed (*n* + 1)th case. Let us refer to the forgotten case as *c*_*frg*_. The inactivity of cases may be attributed to the sparse event distribution of cases, but the criteria for inactivity do not take into consideration such factors. At some point in the future, an orphan event *o* in the perspective of *c*_*frg*_ is observed on the stream, i.e., *π*_*c*_(*o*) = *c*_*frg*_. The orphan event *o* has lost its trace prefix, and hence, its corresponding case will be referred to as the *prefix-missing* case. To store this orphan event *o* belonging to *c*_*frg*_, a space has to be freed up by forgetting a (supposedly) inactive case out of the *n* cases in *D*_*C*_. This forgotten case may potentially be in a marking *M* ≠ *M*_*f*_, thereby resulting in an additional *prefix-missing* case. It is important to mention here that not every single observed event may necessitate forgetting a case. Due to varying event distribution, a sequence of observed events may belong to the cases already existing in *D*_*C*_.

During conformance checking of the orphan event *o*, the prefix-alignments will treat the case corresponding to the orphan event *π*_*c*_(*o*) = *c* as an altogether new case starting with activity *a*, i.e., *π*_*a*_(*o*) = *a*. Since an orphan event does not correspond to any of the activities represented by the transitions enabled in the initial marking of the process model *T*_*ini*_, the prefix-alignments will mark this orphan event as a log move and hence its corresponding case as non-conformant. The case and accordingly its alignment may evolve with observing any further events of *c*_*frg*_. The exact nature of the evolving alignment depends on multiple factors, for instance, the length of the forgotten trace, the length of the observed trace, and the possible orientation in the process model.

Table 2 demonstrates for an example trace some possible combinations of a forgotten trace, events observed after forgetting, and subsequent prefix-alignments with the process model of Figure 2. In general, an orphan event *o* and its succeeding events will be marked as log moves (as they are less costly) until we get a sequence of events where the sum of the costs of the model moves for the missing prefix and synchronous moves for the observed sequence of events is less than the cost for declaring the whole observed sequence of events as log moves. For instance, consider the second row of Table 2 where ⟨*A*, *B*⟩ is the forgotten trace for a case. An orphan event *C* for this case is observed. Considering a unit cost of 1.0 for model and log moves, declaring the orphan event as a log move has a raw trace fitness cost of 1.0, while positioning it as a synchronous move, i.e., ⟨(≫, *A*), (≫, *B*), (*C*, *C*)⟩, has a raw trace fitness cost of 2.0; therefore, it will be declared as a log move. Upon observing the event *D* for the same case makes the observed sequence of events as ⟨*C*, *D*⟩, but the prefix-alignments still declare these two events as log moves. If the next observed event happens to be *G*, then the prefix-alignments declare the sequence ⟨*C*, *D*, *G*⟩ as synchronous moves with model moves for *A* and *B*. In the example, although conformant by origin, the case is still penalized due to the missing prefix caused by the inactivity-based forgetting mechanism.

**TABLE 2:** Example prefix-alignments for case “7” of event log of Table 1 with inactivity-based forgetting at different prefix lengths.

Forgotten trace	Orphan event(s)	Prefix-alignments
⟨A⟩	⟨B⟩	⟨(B, ≫)⟩
	⟨B, C⟩	⟨( ≫, A), (B, B), (C, C)⟩
⟨A, B⟩	⟨C⟩	⟨(C, ≫)⟩
	⟨C, D⟩	⟨(C, ≫), (D, ≫)⟩
	⟨C, D, G⟩	⟨( ≫, A), ( ≫, B), (C, C), (D, D), (G, G)⟩
⟨A, B, C⟩	⟨D⟩	⟨(D, ≫)⟩
	⟨D, G⟩	⟨(D, ≫), (G, ≫)⟩
	⟨D, G, H⟩	⟨(D, ≫), (G, ≫), (H, ≫)⟩

In the example mentioned in the previous paragraph, we assumed the trace observed after forgetting the prefix to be completely conformant. Consider again the second row of Table 2 but this time with the observed events ⟨*C*, *H*, *I*⟩ after forgetting ⟨*A*, *B*⟩. In this scenario, the prefix-alignments will treat all the observed events as log moves ⟨(*C*, ≫), (*H*, ≫), (*I*, ≫)⟩ with a raw trace fitness cost of 3.0, instead of maximally synchronizing them ⟨(≫, *A*), (≫, *B*), (*C*, *C*), (≫, *D*), (≫, *G*), (*H*, *H*), (*I*, *I*)⟩ with a cost of 4.0. Depending on the distribution of the events, some cases may possibly be forgotten more than once, thereby increasing their probability to be maximally reported as non-conformant. For instance, the cases in the blue-colored box of the dotted chart of Figure 5 are probable candidates for multiple forgetting due to their sparse distribution of events.

We highlighted the missing-prefix problem in the perspective of a single case. In an event stream and *n*-bounded *D*_*C*_, every (*n* + *k*)th distinct case where 1 ≤ *k* ≤ *∞* will cause a case in *D*_*C*_ to be forgotten. Depending on the number of parallel running cases and their event distribution, multiple (and most probably distinct) cases will suffer from the missing-prefix problem. Consider a worst-case scenario. At any point in time, at least *n* + *k* where *k* ≥ 1 distinct cases are executing in a round-robin fashion such that the *i*th event for any of these *n* + *k* cases is observed only after the (*i* − 1)th event has been observed for all of these cases. In this particular scenario, after observing the first event for all the *n* cases, every subsequent observed event will imply forgetting a case out of *D*_*C*_ to accommodate the case corresponding to the last observed event. Assuming all the cases to be starting with an activity corresponding to one of the transitions in *T*_*ini*_, except the first event for all these cases, any other event will be reported as a log move.

Our proposed solution tackles the missing-prefix problem through two inter-twined steps: prefix imputation and impactful forgetting mechanism.

**Algorithm 1:** Model-based prefix imputation.

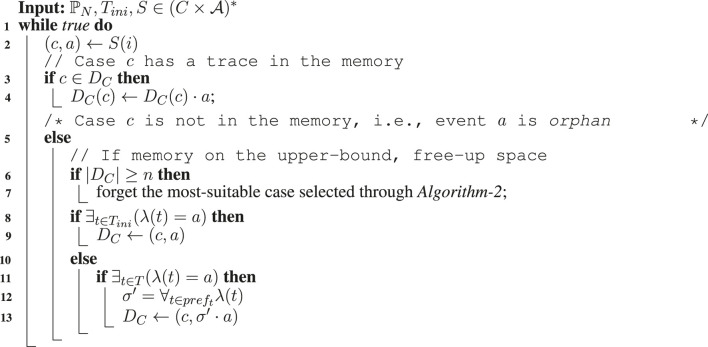

### 4.2 Prefix Imputation

Consider again the state of the event stream processing system we sketched in the last section where *n* distinct cases have already been observed and accordingly stored in *D*_*C*_. An event (*c*, *a*) belonging to a case other than the already stored *n* cases, i.e., *c* ∉ *D*_*C*_, is observed. Let us refer to this case as the (*n* + 1)th case. The forgetting mechanism of our proposed approach, to be detailed in the next section, systematically forgets one of the cases stored in *D*_*C*_ to free up space for the recently observed (*n* + 1)th case. In short, for forgetting cases, our forgetting mechanism gives priority to the cases whose current marking *M* can most probably be reproduced later on observing the orphan event for the case. Let us refer to the forgotten case as *c*_*frg*_. Imagine, at some point in the future, an orphan event *o* in the perspective of *c*_*frg*_ is observed on the stream, i.e., *π*_*c*_(*o*) = *c*_*frg*_. The orphan event *o* has lost its trace prefix, and hence, its corresponding case will be referred to as the *prefix-missing* case. To store this orphan event *o* belonging to *c*_*frg*_, a space has to be freed up by forgetting a suitable case out of the *n* cases in *D*_*C*_, and this cycle continues.

As per our proposed approach, before subjecting the prefix-missing case to conformance checking, its orphan event *o* is positioned in the process model *N*. The activity represented by the orphan event, i.e., *π*_*a*_(*o*) = *a*, is mapped to a relevant transition *t* ∈ *T* in the process model *N* such that *a* = *λ*(*t*). Next, a shortest prefix *pref*_*t*_ for transition *t* is calculated as per the process model *N*. The *pref*_*t*_ is transformed into the alphabet Λ and imputed as a prefix to *a* to reproduce the trace for *c*_*frg*_. Subjected to conformance checking, although the prefix-alignments will treat this trace as corresponding to an altogether new case, the case will not be wrongly penalized. Algorithm 1 provides an algorithmic summary of our proposed approach. Upon observing a stream event (*c*, *a*), the approach (lines 3–9) makes sure if the event is an orphan *o*. If found to be an orphan, the approach positions the activity *a* in the process model (line 11) and accordingly imputes the event with a suitable prefix *pref*_*t*_ (line 12).

Table 3 demonstrates some example scenarios. A trace is forgotten at different prefix lengths and accordingly imputed with a suitable trace prefix by our proposed approach. The prefix-alignment of the imputed traces with the process model of Figure 2 highlights the efficacy of our approach. For instance, consider again the second row of Table 3 where ⟨*A*, *B*⟩ is the forgotten trace. An orphan event *C* for this forgotten case is observed. Our approach imputes the orphan event with the prefix ⟨**A**, **B**⟩ guided by the process model of Figure 2 (the imputed prefix is represented in bold). The resulting trace ⟨**A**, **B**, *C*⟩ is fully conformant and bears a raw trace fitness cost of 0.0. The future alignment of this case mostly depends on the future events. For instance, after observing the orphan event *C* and accordingly imputing it with the prefix ⟨**A**, **B**⟩, we observe the sequence of events ⟨*H*, *I*⟩ for this case. The prefix-alignments will result in a prefix-alignment of ⟨(*A*, *A*), (*B*, *B*), (*C*, *C*), (*H*, ≫), (*I*, ≫)⟩ with a raw trace fitness cost of 2.0, in comparison with a prefix-alignment ⟨(≫, *A*), (≫, *B*), (*C*, *C*), (≫, *D*), (≫, *G*), (*H*, *H*), (*I*, *I*)⟩ by the competitor approach with a cost of 4.0.

**TABLE 3:** Example prefix-alignments for case “7” of event log of Table 1 with our proposed imputation approach and forgetting at different prefix lengths. The imputed prefix for each trace is represented in bold.

Forgotten trace	Orphan events	Trace with prefix imputation	Prefix-alignments
⟨A⟩	⟨B⟩	⟨**A**, B⟩	⟨(A, A), (B, B)⟩
	⟨B, C⟩	⟨**A**, B, C⟩	⟨(A, A), (B, B), (C, C)⟩
⟨A, B⟩	⟨C⟩	⟨**A**, **B**, C⟩	⟨(A, A), (B, B), (C, C)⟩
	⟨C, D⟩	⟨**A**, **B**, C, D⟩	⟨(A, A), (B, B), (C, C), (D, D)⟩
	⟨C, D, G⟩	⟨**A**, **B**, C, D, G⟩	⟨(A, A), (B, B), (C, C), (D, D), (G, G)⟩
⟨A, B, C⟩	⟨D⟩	⟨**A**, **B**, **C**, D⟩	⟨(A, A), (B, B), (C, C), (D, D)⟩
	⟨D, G⟩	⟨**A**, **B**, **C**, D, G⟩	⟨(A, A), (B, B), (C, C), (D, D), (G, G)⟩
	⟨D, G, H⟩	⟨**A**, **B**, **C**, D, G, H⟩	⟨(A, A), (B, B), (C, C), (D, D), (G, G), (H, H)⟩

Our proposed approach is able to deal with the missing-prefix problem for every (*n* + *k*)th distinct case observed on the event stream, where *k* ≥ 1, subject to the condition that a suitable trace, i.e., being reproducible as a prefix, is available in *D*_*C*_ at the moment of observing the first event of that (*n* + *k*)th case. With a stream fulfilling the mentioned criteria, the number of in-parallel running cases has a limited impact on our proposed approach. Event distribution and the different noise associated with events are critical factors influencing the correctness and the quality of our imputed prefixes, as will be realized through the experimental evaluation.

Positioning an orphan event in the process model, a fundamental step in our proposed approach, may not always be deterministic due to the reasons discussed in the next section.

#### 4.2.1 Non-Determinism in Prefix Imputation

Determinism in prefix imputation refers to the situation where the marking *M* at which a case is forgotten can be reproduced with certainty once an orphan event *o* for the forgotten case is observed. On the contrary, non-determinism in prefix imputation refers to the situation where uncertainty exists regarding the marking of the forgotten case and the marking which can be reproduced through prefix imputation once an orphan event for the forgotten case is observed.

Apparently simple and just a combination of limited places, transitions, and arcs, Petri net–based process models are behaviorally quite complex artifacts. This embedded behavioral complexity of Petri nets is one of the major contributors to non-determinism in our prefix-imputation approach that demands some heuristic decisions during the imputation step. Concurrency, looping, or a combination of these constructs is by far the most complex phenomenon for our prefix-imputation approach.

For instance, consider the AND-split pattern after transition *t*_7_ in Figure 2. This transition has three output places, i.e., *t*_7_• = {*p*_6_, *p*_7_, *p*_8_}, which are accordingly leading to three parallel branches. Although this process model has a single transition in each parallel branch, other business process models may possibly have multiple transitions in each parallel branch. Suppose an orphan event corresponding to the activity represented by the transition *t*_10_ is observed, which implies that *t*_7_ has already been fired and, as a result, three tokens were produced in its three output places. While the orphan event corresponding to transition *t*_10_ deterministically hints at the position of the token in place *p*_8_, the location of the rest of the two tokens is still uncertain. The set of *non-deterministic markings* includes {[*p*_6_, *p*_7_, **p**_**8**_], [*p*_7_, **p**_**8**_, *p*_9_], [*p*_6_, **p**_**8**_, *p*_10_], [**p**_**8**_, *p*_9_, *p*_10_]}. This uncertainty may gradually decrease with the evolution of the trace. Events corresponding to the activities represented by the transitions in the other two parallel branches are observed, thereby making the position of the other two tokens deterministic. Alternatively, the event corresponding to the activity represented by transition *t*_11_ is observed which is basically the end of an AND-join pattern and synchronizes the tokens in all the three parallel branches. The latter scenario also makes the situation deterministic, by requiring the three tokens to be in the three input places of *t*_11_, i.e., {*p*_9_, *p*_10_, *p*_11_}. For convenience, a cluster of related non-deterministic transitions is referred to as a *non-deterministic region*. For instance, transitions *t*_8_, *t*_9_, and *t*_10_ in the process model of Figure 2 constitute a non-deterministic region.

Due to the highlighted problem, our forgetting mechanism least favors the cases having a non-deterministic current marking. Nevertheless, in some situations, such cases may be the only good candidate(s) to be forgotten. We, therefore, devise a token displacement technique to cope with the uncertainty regarding the tokens in non-deterministic regions of a process model. Once an orphan event *o* for such a case is observed, initially we only impute a shortest prefix *pref*_*t*_ which at least enables the transition bearing the label corresponding to the activity represented by the orphan event. For instance, upon observing an orphan event corresponding to transition *t*_10_, we impute it with the prefix ⟨**A**, **B**, **C**, **D**, **G**⟩. Next, if we observe any event corresponding to transition *t*_8_ or *t*_9_, the token position in their respective branches becomes deterministic. Upon receiving an event corresponding to transition *t*_11_, we check if any branch exists where the token position is still non-deterministic. If so, we keep firing the enabled transitions in those branches to displace the tokens at their input places to the respective input places of *t*_11_, resulting in a shift from a non-deterministic state to a deterministic one.

In some Petri net process models, multiple transitions either bear identical labels or semantically refer to the same activity of the process, the concept referred to as *label duplication*. Dealing with duplicate labels is also non-deterministic as multiple transitions are candidates for mapping to the orphan event. We adapt a heuristic approach to deal with duplicate labels and map the orphan event to the transition bearing the shortest prefix *pref*_*t*_ in comparison with the other candidates. The rationale behind this heuristic approach is the fact that if a shortest imputed prefix *pref*_*t*_ turns out to be incorrect, it will probably contribute less to the wrongly estimated trace fitness costs.

**Algorithm 2:** Searching suitable cases for forgetting.

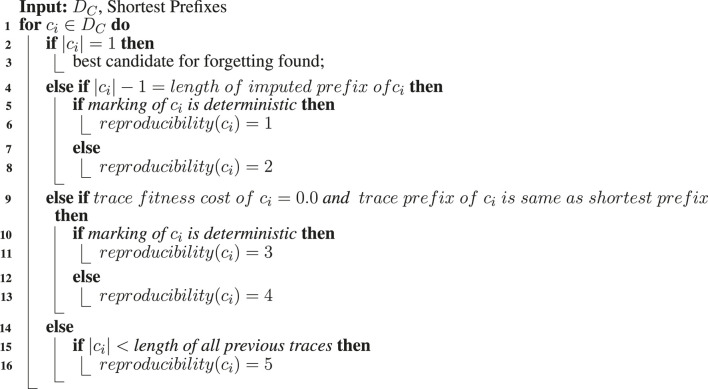

### 4.3 Impactful Case Forgetting

As discussed in the previous section, not all forgotten cases and their respective markings *M* can be reproduced with certainty upon observing their orphan events *o*. Therefore, we devise an impactful forgetting mechanism that categorizes the cases existing in *D*_*C*_ according to their reproducibility score. Accordingly, the case having the least uncertainty about its reproduction is forgotten. The devised mechanism increases the likelihood of correct imputation decisions, thereby providing leverage to the imputation step discussed in the previous section. Algorithm 2 summarizes our single-pass categorization mechanism. The following steps briefly explain the various conditions to determine the reproducibility scores of the cases in *D*_*C*_:

1) The first condition (line 2) is related to the length of traces. A monuple (singleton) trace, i.e., the one consisting of a single event, is the most ideal candidate as it by default belongs to *T*_*ini*_ and hence can be reproduced with high certainty.
2) The second condition (line 4) takes into account the imputation history of the cases in *D*_*C*_. Cases with an imputed prefix and having their current marking being unchanged after the imputation can be reproduced with certainty. The latter part of the condition in essence implies that no further events have been observed after the imputation, and hence, the marking can be reproduced. With reference to the discussion in the previous section, cases with a deterministic current marking are preferred over the cases with a non-deterministic current marking (lines 5–8).
3) The third condition (line 9) is related to the conformance, i.e., trace fitness costs of the cases, and their resemblance to the corresponding shortest prefix as per the reference process model. First, we check if a trace is completely conformant, i.e., having a raw trace fitness cost of 0.0. Then, we check if the *n* − 1 length trace prefix of the length *n* trace is the same as the shortest prefix of the transition corresponding to the activity of the event at position *n* in the trace. If both checks are fulfilled, then the trace can be reproduced with certainty. Again, cases with deterministic current marking are preferred over the cases with non-deterministic current marking (lines 10–13).
4) Concomitantly, we keep track of the case with the minimum trace length in *D*_*C*_ (lines 14–16).

Ideally, we look for a monuple trace, and if found, further categorization is not required. Otherwise, we keep traversing *D*_*C*_ and labeling the cases with a reproducibility score on the basis of the mentioned conditions. After completely traversing *D*_*C*_, we select the case with the maximum certainty regarding its reproduction, i.e., minimum reproducibility score as per the above-mentioned conditions. If a suitable candidate is not found, then the case with the minimum trace length in *D*_*C*_ is selected to be forgotten with the rationale that a wrong prefix imputation for its orphan event will cause the least damage, i.e., wrongly estimated non-conformance. It is important to mention that we make an assumption about the orphan event to be legitimate and noise-free. This basic assumption bridges the two steps of our proposed approach. Noisy events observed as orphan events can negatively affect the quality of prefix imputation, as observed in the experimental evaluation.

## 5 Evaluation

In this section, we discuss the implementation of our proposed approach followed by the details on the experimental setup, the purpose, and the results of the conducted experiments.

### 5.1 Implementation

The proposed approach is evaluated through a prototype implementation^1^ on top of the PROM^2^ framework. The prototype requires a Petri net process model *N* and its initial and final markings *M*_*i*_, *M*_*f*_ as input. An oracle, be it an expert or algorithm, marks the non-deterministic regions in the process model. To keep the run-time computations minimal, all the process model–related computations are performed a priori in an offline set-up phase, for instance, the calculation of the shortest prefixes. The prototype uses the prefix-alignments approach of van Zelst et al. (2019) for conformance checking. The experiments are conducted on a standard machine installed with Windows 10 64 bit, an Intel Core i7-7700HQ 2.80 GHz CPU, and 32 GB of RAM.

### 5.2 Experimental Setup

Process models and event data, the two first-class citizens of conformance checking, are quite diverse artifacts. The complexity of process models ranges from a simple sequence of activities to a combination or nesting of loops, choices, and concurrency, with duplicate labels as an add-on. The degree of these constructs is also directly related to the complexity of a process model. Event data can be diverse on the level of individual events and on the case level. Events within a case can have a single type or multiple types of noise, such as missing, redundant, or dislocated (out-of-order) events associated with them. On a higher level, multiple cases can be running in parallel. The efficacy of our proposed approach, in terms of the quality or accuracy of the predicted prefixes and accordingly the resulting estimated conformance statistics, is correlated with the aforementioned dynamics of the process models, events, and cases. The following experiments are designed such that the impact of these three types of complexity sources on our proposed approach, either in isolation or in combination, can be discovered. The foremost criteria to select event logs for these experiments are the arrival rate of new cases such that the number of cases running in parallel cases surpasses our memory limits. We briefly explain the purpose of each type of experiment in Section 5.3.

Table 4 provides the general details of the event data used in the experiments. For generating the synthetic event data, we modeled and simulated the normative process model of the Conformance Checking Competition 2019 (CCC’19) event log (Munoz-Gama et al., 2019) in CPN Tools.^3^ In line with the purpose of the experiment with synthetic event data, no noise is introduced during the simulation to keep all the cases 100% conformant with the process model. The event distribution of this synthetic event log is provided as a dotted chart in Figure 5. We control the arrival rate of cases such that a surge in cases (highlighted in the red-colored box) causes the number of in-parallel running cases to surpass the memory bound. In all our experiments, we are using *n* = 100 as an upper bound on the number of cases allowed to be stored simultaneously in *D*_*C*_, unless stated otherwise. As visible in the dotted chart of Figure 5, along with the cases initiated in the surge period, the pre-surge cases located in the blue-colored box are still active.

**TABLE 4:** Details of the event data used in the experimental evaluation.

Event log	Cases	Events	Event classes	Remarks
CCC’19	300	7,800	29	Synthetic event log
BPIC’12 application process	13,087	60,849	10	Real event data
BPIC’12 application and offer processes	13,087	92,093	17	Real event data
Road traffic fine management process	150,370	561,470	11	Real event data

The second and third event logs belong to Business Process Intelligence Challenge (BPIC’12) (van Dongen, 2012). These real event data belong to a Dutch Financial Institute regarding the applications for personal loans or overdraft within global financing organization data. The process consists of a backbone *application* process and its integral *offer* sub-process. The *application* process bears some distinguishing characteristics in isolation and in combination with the *offer* sub-process. Therefore, we analyze the application event data individually as well as in combination with the offer event data as a holistic process. The normative process models used in these experiments have been developed by a business process modeling expert in consultation with the domain knowledge experts from the financial institute. These process models are provided in the Supplementary Material. The fourth event log consists of real event data extracted from an information system responsible for managing road traffic fines (de Leoni and Mannhardt, 2015). The process model used in the experiment has been discovered from the event data by a process mining expert, with further enrichment through the domain knowledge and information regarding traffic regulations. The mentioned process model is provided in the Supplementary Material.

We realize an event stream by dispatching events in a log one at a time on the basis of their actual timestamps so that the case and event distribution of the event log is preserved. We use the default unit cost of 1.0 for both log and model moves, while synchronous and silent transition *τ* model moves incur a cost of 0.0. The non-zero costs of all the individual log and model moves for a trace are combined to calculate the *raw trace fitness cost*. A zero raw trace fitness cost indicates that the trace is fully conformant with the reference process model, while a non-zero cost hints at some non-conformant behavior in the case. The magnitude of the cost indicates the level of non-conformance, such that a lower trace fitness cost, as a result of a high number of synchronous moves in the optimal alignment, indicates higher conformance of the trace with the reference process model.

### 5.3 Results

We evaluate our approach with respect to combinations of varying complexities of the process model, events, and cases. Our aim is to answer questions regarding different quality aspects of our proposed approach. Through our first experiment with the noise-free synthetic event log CCC’19, we answer the following question in Section 5.3.1: *“Can our approach perform as good as an offline approach bearing unlimited memory with noise-free event data?”* Related to the robustness of our proposed approach, the next set of experiments with real event data of BPIC’12 investigates in Section 5.3.2: *“How do different types of noise, in isolation and in combination, together with process models of complex constructs affect the quality of our prefix-imputation decisions?”*. Our final experiment with the real event data of the road traffic fine management process evaluates the scalability of our approach in Section 5.3.3 through validating our claim that *“The number of cases running in parallel has a limited impact on our approach”*.

#### 5.3.1 Synthetic Data Study

In our first experiment, we ascertain the peak accuracy of our proposed approach with the CCC’19 synthetic event log and its very straightforward process model. The relevant process model is quite sequential and does not bear any complex constructs. Similarly, all the cases in the event data are noise-free and fully compliant with the relevant process model. As depicted in the dotted chart of Figure 5 of these event data, the cases are diversely distributed and a single surge of in-parallel running cases is simulated in order to surpass the memory limit of *n* = 100. The surge necessitates forgetting in-parallel running cases. The majority of the cases initiated during the surge are short-lived so that we can observe the post-surge effect as well.

We perform the experiment with the streaming version of prefix-alignments of van Zelst et al. (2019) without prefix imputation, and then with the same streaming version of prefix-alignments of van Zelst et al. (2019) with our proposed prefix-imputation approach in place. The prefix-alignments without prefix imputation forget cases in overflow situations on the basis of *inactivity* criteria, and therefore, this configuration is considered the competitor. In contrast, our prefix-imputation approach uses the proposed impactful forgetting mechanism.

**FIGURE 6:**
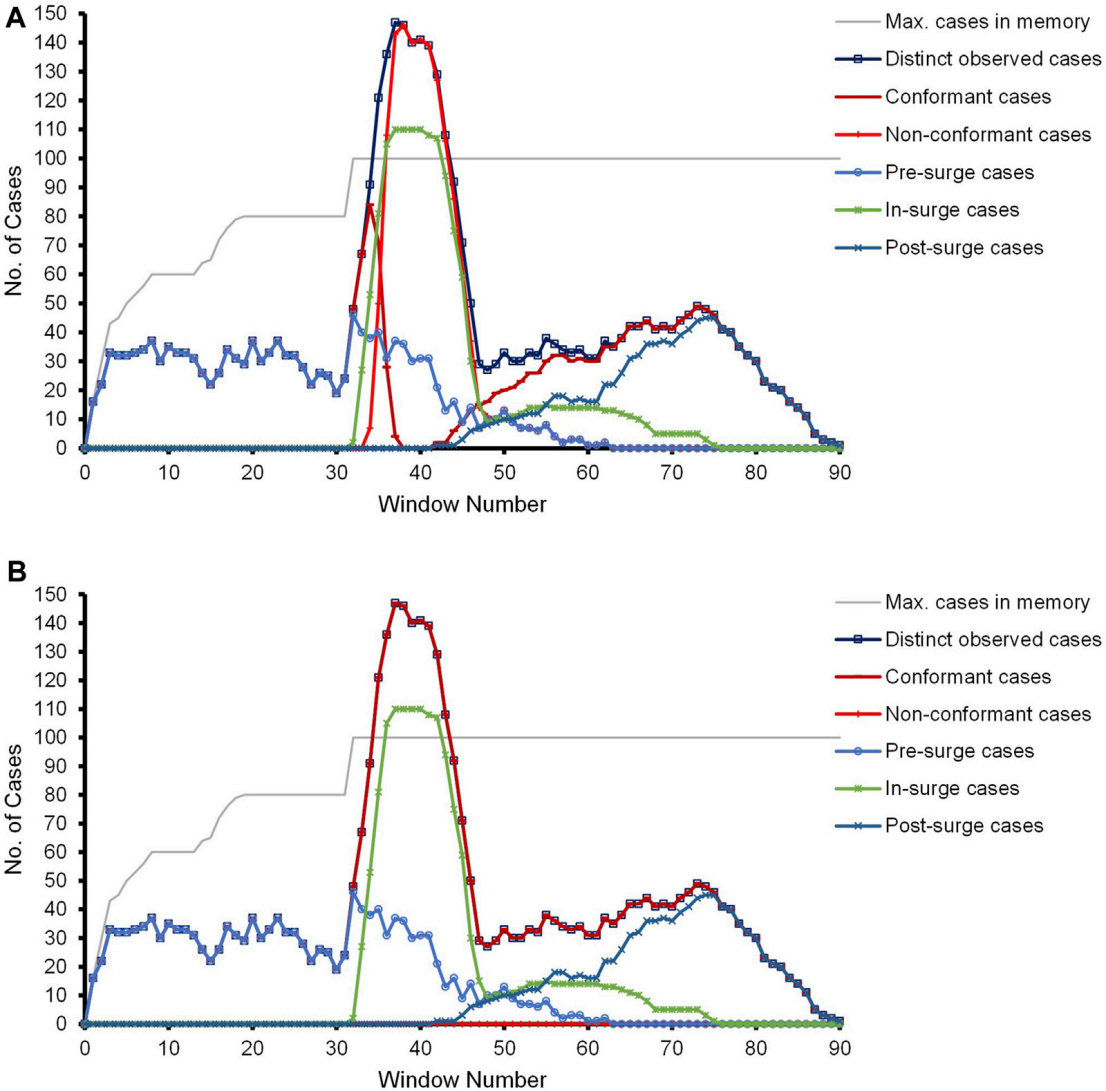
Results of the experiment with the CCC’19 synthetic event data. **(A)** Prefix-alignments without our prefix-imputation method. **(B)** Prefix-alignments with our prefix-imputation method.

Due to the event and case distribution and to clearly observe the pre-surge, in-surge, and post-surge effects, we are reporting different statistics in terms of the number of cases within different windows. Events belonging to the same date are reported collectively as one window. For each window, we present the maximum number of cases in memory, the distinct observed cases, the conformant and non-conformant cases, and the categorization of the observed cases with respect to their arrival in the context of the surge as pre-surge, in-surge, or post-surge. Figure 6A depicts the results of the competitor approach. Correlating the dotted chart of Figure 5 of the CCC’19 event data and the experimental results in Figure 6A, a surge of (short-life) cases starts at Window 32 such that the number of in-parallel running cases surpasses the memory bound of *n*. The surge eventually diminishes at Window 43.

With the number of in-parallel running cases surpassing the memory bound, the pre-surge and even some in-surge cases are forgotten out of *D*_*C*_ to accommodate subsequent cases. This cycle continues till the influx of new cases and already running cases stabilizes at or below the limit *n*. As the orphan events of the forgotten pre-surge and in-surge cases are missing their prefixes, starting at Window 33, the competitor approach reports them as non-conformant. Depending on the event distribution of the cases running in parallel, the curves of the distinct and non-conformant cases coincide in some of the windows. The (improper) penalization of prefix-missing cases even continues post-surge till such cases are active. Once these prefix-missing cases reach their final marking, the effect of missing-prefix problem diminishes. Referring to Figure 6A, at Window 64 onward, only the post-surge and some of the in-surge cases are active, and hence, no non-conformance is observed.

Figure 6B depicts the results of our proposed prefix-imputation approach. As evident, the prefix imputation handles the surge by wisely forgetting cases and then reproducing them as the missing prefix of their orphan events. As a result, the prefix-alignments do not report any incorrect non-conformance during the entire process. As evident in the figure, the curves of the distinct and conformant cases completely overshadow each other. Accordingly, the line of the non-conformant cases is lying on the *x*-axis, which would also be the case with an offline approach bearing unlimited memory.

#### 5.3.2 Robustness Study

Through these experiments, we aim to check the behavior of our approach in response to the different types of noise prevailing in the event data and varying levels of complexity existing in the relevant process models. We use two distinct sets of the real event data of BPIC’12, primarily because of their distinct behaviors. Through the experiment with the event data of the backbone *application* only process of BPIC’12, we are exposing our approach to only *dislocation* or *out-of-order* type of event noise contained in these event data. The relevant process model, provided in the Supplementary Material, is relatively simple and equipped with all sorts of constructs, except for looping and label duplication. The steady arrival of fresh cases results in the number of in-parallel running cases to surpass the memory limit of *n* = 100.

Through the experiment with the event data considering both the backbone *application* process and its integral *offer* sub-process of BPIC’12, we expose our approach to multiple types of event noise and on the contrary to a high-level complex process model. Cases in this event log contain missing and out-of-order types of event noise. Some cases even contain both of these types of noise. The reference process model, provided in the Supplementary Material, bears all the characteristics of a complex model. This model has a high degree of label duplication, such as four transitions sharing the label A_DECLINED. Additionally, the model is not sound (Van Der Aalst, 2016). Majority of the transitions belong to a non-deterministic concurrent region. The non-deterministic region is quite complex by virtue of nested loops, choices, and concurrency. The arrival rate of fresh cases is consistent such that the number of in-parallel running cases surpasses the memory limit of *n* = 100.

The experiments focus on the comparison of the conformance estimation of the streaming version of prefix-alignments of van Zelst et al. (2019) with and without our proposed prefix-imputation approach in place against that of the non-streaming version of prefix-alignments of PROM (to be treated as the ground-truth). Both the non-streaming version of prefix-alignments of PROM and the streaming version of prefix-alignments of van Zelst et al. (2019) rely on the A^⋆^ shortest path algorithm (Hart et al., 1968) for searching optimal alignments *γ*_*opt*_. The key difference between the two approaches is related to their suitability for different environments. The former is an offline approach that is well-suited for the analysis of historic event data, while the latter is optimized to be efficient in streaming environments. The prefix-alignments without prefix-imputation configuration (competitor approach) forget cases in overflow situations on the basis of *inactivity* criteria, while our prefix-imputation approach uses the impactful forgetting mechanism. The steady increase of fresh cases results in the number of in-parallel running cases to be greater than the memory limit. For this reason, we use in the following the number and the cumulative raw trace fitness cost of all non-conformant cases at different prefix lengths as our comparison metrics.

**FIGURE 7:**
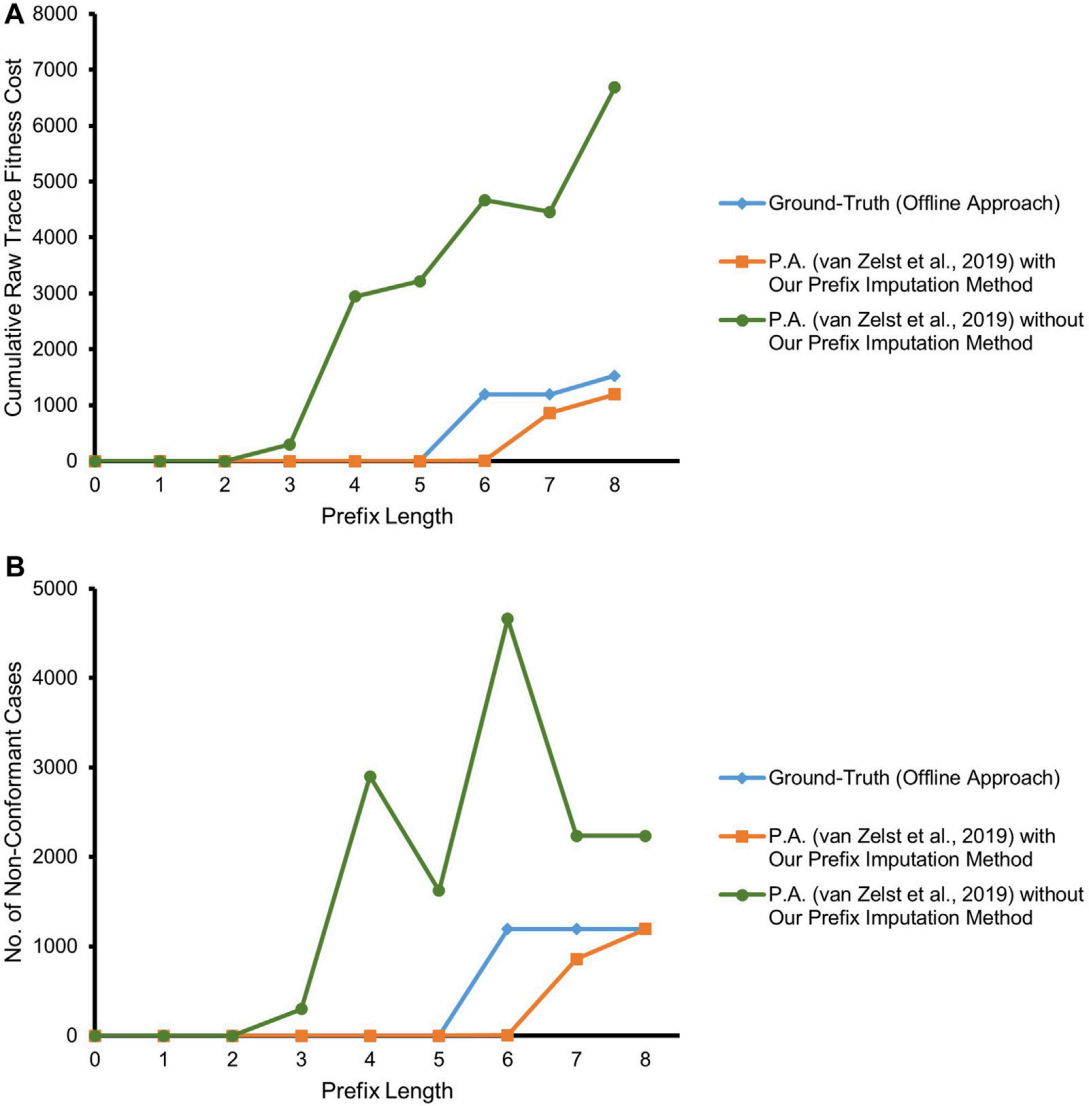
Prefix-alignments with and without our prefix-imputation method for the *application* process of the BPIC’12 event data. **(A)** Cumulative raw trace fitness cost. **(B)** Number of non-conformant cases.

**FIGURE 8:**
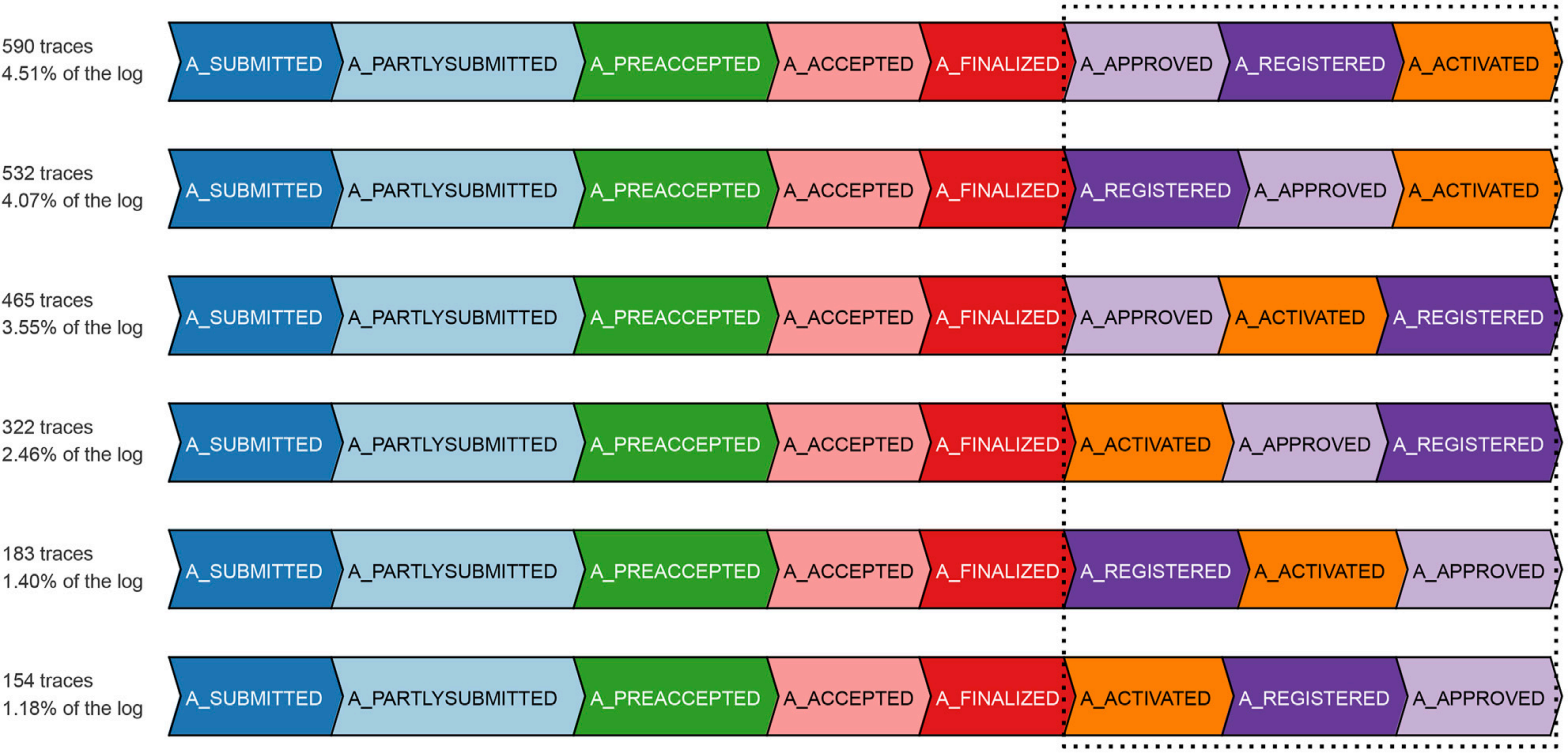
Cases with a prefix length of at least 6 in the *application* process of the BPIC’12 event data.

Figure 7 depicts the result of our experiment with the real event data of BPIC’12, taking into account only the backbone *application* process and its related events. The competitor approach starts deviating from the ground-truth by overestimating non-conformance right at a prefix length of 3, as it highly suffers from the missing-prefix problem. This overestimation keeps increasing almost linearly with the increasing prefix length. Our proposed approach and the ground-truth are in complete agreement for all the cases till prefix length 5. Starting from prefix length 6, there is a surprising disparity between the ground-truth and our proposed approach. Our approach underestimates non-conformance for some of the cases. We investigate the cases with at least a prefix length of 6, in light of the reference process model. As depicted in Figure 8, all such cases bear the same set of activities {A_APPROVED, A_REGISTERED, A_ACTIVATED} at their last three trace indices, but in a random order such that these three activities appear to be executed in parallel, while the relevant AND-split pattern of the process model allows only {A_REGISTERED, A_ACTIVATED} to be executed in parallel after the execution of A_APPROVED.^4^

The ground-truth (always having the complete and true prefix) treats the events not conforming to the mentioned pattern of the process model as out-of-order arrived events and reports the cases as non-conformant. Especially, the cases with two-step swapping where A_APPROVED is observed after {A_REGISTERED, A_ACTIVATED} are penalized the most with a raw trace fitness cost of 2.0, by considering both A_REGISTERED and A_ACTIVATED as log moves. On the contrary, as per our proposed approach, the traces at a prefix length of 5 are considered good candidates to be forgotten. Therefore, some of the cases having the mentioned out-of-order event noise are forgotten in case of memory overflow. While observing one of the {A_REGISTERED, A_ACTIVATED} out-of-order events as an orphan event, our approach imputes a prefix including A_APPROVED, and therefore, the out-of-order noise of such cases gets masked at prefix length 6. Once the swapped A_APPROVED event is observed for such a case, it is reported as a log move, and the trace gets a raw trace fitness cost of 1.0, which is an underestimation in comparison with the cost of 2.0 of the ground-truth. It may however be noted that, at prefix length 8, which is the last event of the relevant cases, the number of cases reported to be non-conformant by our approach resembles the ground-truth.

**FIGURE 9:**
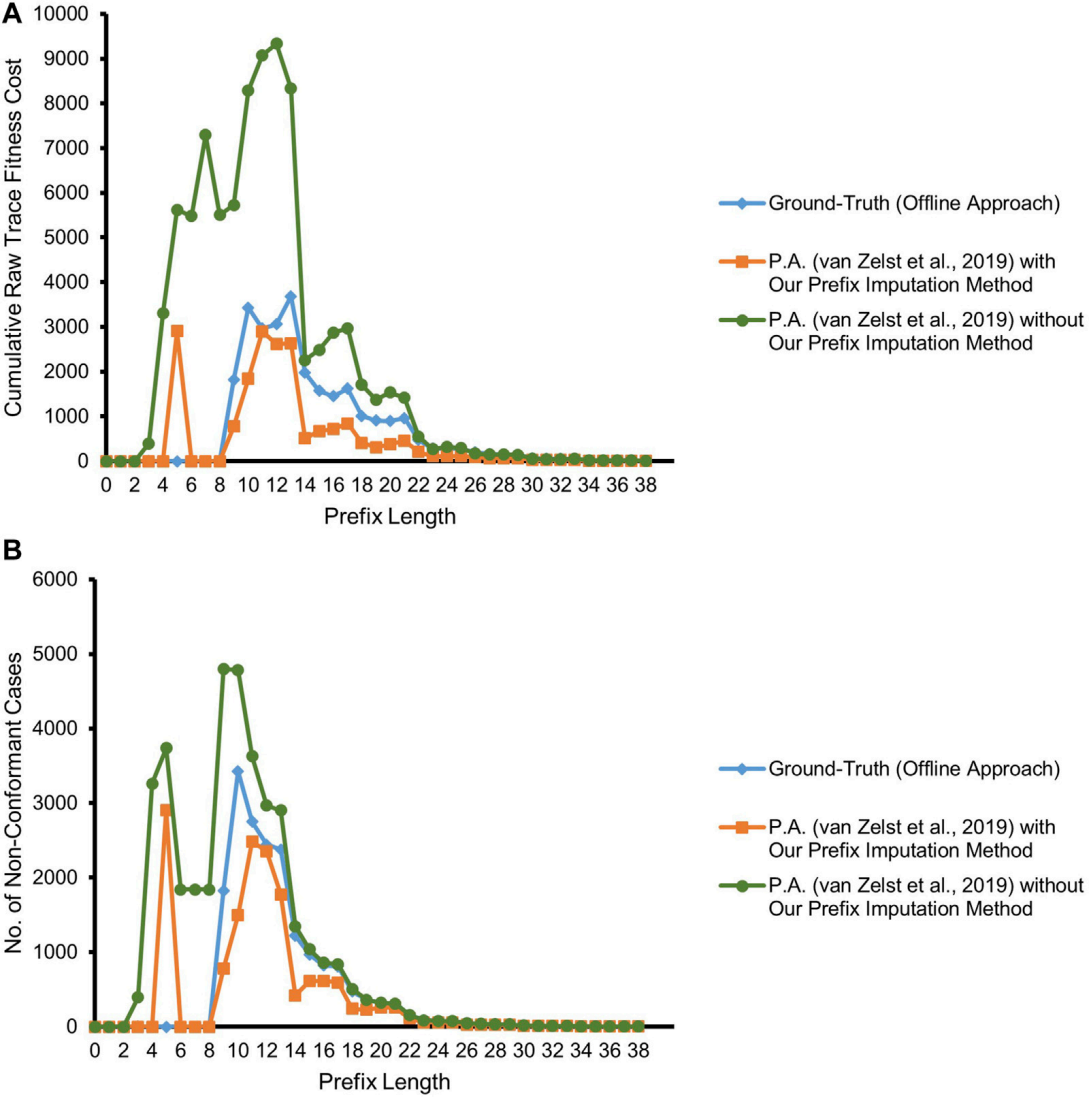
Prefix-alignments with and without our prefix-imputation method for the *application* process and its *offer* sub-process of the BPIC’12 event data. **(A)** Cumulative raw trace fitness cost. **(B)** Number of non-conformant cases.

Figure 9 refers to the results of our experiment with the real event data considering both the backbone *application* process and its integral *offer* sub-process of BPIC’12. As illustrated in the figure, the competitor approach starts deviating from the ground-truth by overestimating non-conformance right at prefix length 3 due to suffering from the missing-prefix problem. Interestingly, the approach is almost in agreement with the ground-truth at prefix length 14. The reason for this unexpected agreement lies in the fact that the majority of the cases in the event log have a non-fitting (noisy) event at prefix length 14 which are marked as non-conformant by the ground-truth. Due to the missing-prefix problem, the competitor approach also marks almost all of the cases at this prefix length as non-conformant and hence in agreement with the ground-truth.

In contrast, referring to Figure 9, our proposed approach in most of the cases is in agreement with the ground-truth, except for some cases in two regions. At prefix length 5, non-conformance is overestimated, and starting at prefix length 9, it is underestimated for some of the cases. Investigating the cases having at least a prefix length of 5, along with taking into consideration the reference process model, a combination of a silent transition leading to an AND-split pattern and a duplicate label bearing transition as its predecessor, causes the prefix-alignments to find an optimal alignment for such traces only at prefix length 6.

**FIGURE 10:**
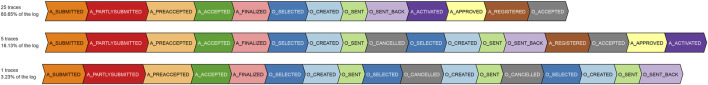
Example cases with different noise types in the *application* process and *offer* sub-process of the BPIC’12 event data.

The investigation of underestimated non-conformance starting at prefix length 9 reveals the *masking* of the different types of noise prevailing in the event data as the major cause. These different types of noise include missing events, out-of-order events, and a combination of both. Only 17% of the cases having a prefix length of 9 or more are fully conformant. Furthermore, 55% of the remaining 83% non-conformant cases are having events with a single noise type, while the events of the remaining 45% bear multiple noise types. Some cases follow a loop in the process model, and hence, multiple instances of the same noise type exist in their traces. Figure 10 depicts one instance of each of the mentioned noise. The noise appears only after prefix length 8, i.e., in the reachable markings of marking [*p*3, *pa*5]. Looking at the markings of the cases forgotten during the course of the experiment, approximately 27% of the cases are forgotten in a reachable marking of [*p*3, *pa*5].

A swap at the input marking of a noisy event gets masked by the prefix imputation, since the noise associated with an orphan event cannot be established in the absence of its prefix. The high percentage of the noisy events existing in the reachable markings of marking [*p*3, *pa*5] and at the same time the large number of forgetting in the reachable markings of [*p*3, *pa*5] increases the likelihood of noisy events being masked by the prefix imputation. On the contrary, the ground-truth, having knowledge of the complete prefix, detects the noise associated with the events and hence correctly estimates the non-conformance.

Along with the underestimation of non-conformance discussed in the previous paragraphs, the label duplication of the process model and our heuristic approach to deal with orphan events bearing duplicate labels cause overestimated non-conformance for a few cases. The orphan events of some of the cases forgotten in the reachable markings of marking [*p*3, *pa*5] are having duplicate labels. For an orphan event with a duplicate label, our heuristic approach maps it to the transition bearing the shortest prefix to be imputed. The mentioned orphan events are therefore mapped to an incorrect duplicate labeled transition, and the events following the orphan events are marked as non-conformant. Such traces, therefore, overestimate the non-conformance in comparison with the ground-truth.

#### 5.3.3 Scalability Study

As a final experiment, we expose our approach to a high degree of in-parallel running cases through the event data of the *road traffic fine management* process. These event data greatly resemble the worst-case scenario we sketched in Section 4. Besides a large number of process instances running in parallel, many types of the process activities are executed in the batch mode. This two-dimensional complexity enables us to stress the forgetting and the imputation steps of our proposed approach. The reference process model, provided in the Supplementary Material, is also behaviorally quite diverse. The majority of the transitions, such as {Payment1, Add penalty}, can be fired an infinite number of times. The activity *Payment* is having label duplication in the process model. Furthermore, the part of the model starting at place *p*4 is *flower-like* (Van Der Aalst, 2016). We adapt the same configuration regarding the competitor approach and the ground-truth as in the previous experiments with real event data of BPIC’12 for this experiment.

**FIGURE 11:**
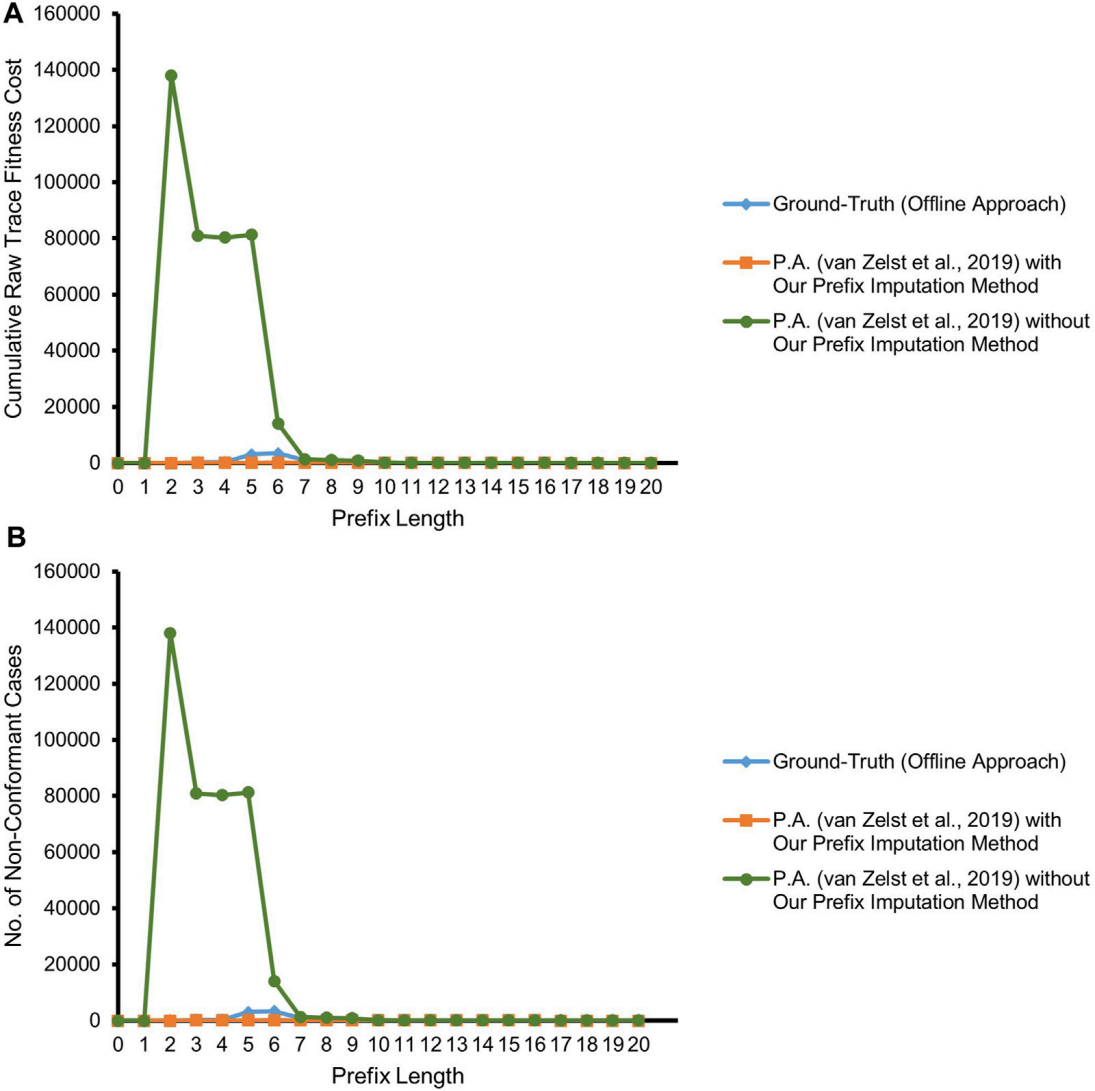
Prefix-alignments with and without our prefix-imputation method for *road traffic fine management* process event data. **(A)** Cumulative raw trace fitness cost. **(B)** Number of non-conformant cases.

As already established in Section 4 and illustrated in Figure 11, the competitor approach reports the first event of all the cases as conformant with synchronous moves. Starting from a prefix length of 2, the majority of the subsequent events are reported as log moves due to the missing-prefix problem. In contrast, our proposed approach finds most of the times a suitable trace, i.e., being reproducible as a prefix, and hence, the curve is almost in agreement with the ground-truth. As in the previous experiment with real event data, some of the cases are having single or multiple non-fitting events. Such noisy events get masked in some of the forgotten cases, and hence, our proposed approach underestimates their non-conformance. This slight disagreement with the ground-truth is visible in Figure 11, especially at prefix lengths of 5 and 6.

### 5.4 Discussion on the Results

We evaluated the efficacy of our proposed approach through the experiments discussed in the previous section. The real event data used in the experiments were selected such that, on the one hand, the reference process models contain a variety of complex behavioral and structural properties and, on the other hand, the event data itself contain almost all sorts of noise, with certain degree of in-parallel running cases.

From the process model perspective, our proposed approach is able to deal with non-determinism caused by behavioral properties like concurrency. The non-determinism caused by label duplication has the potential to negatively affect the quality or correctness of our imputation decisions, resulting in over- or underestimated non-conformance. Another important aspect of the Petri net process models is the distinctiveness of the shortest prefixes. On the one end of the spectrum, we have flower-like process models where almost all the transitions in the process model have the same shortest prefix. For instance, consider the flower-like process model provided in the Supplementary Material where ⟨*A*⟩ is the only shortest prefix for the majority of the transitions in the process model. On the other end of the spectrum, we have *lucent* Petri nets (van der Aalst, 2019) where, by definition, each marking is uniquely identified by the set of enabled transitions, therefore implying distinct shortest prefixes. Lucent Petri net–based process models are best suited for our proposed approach.

As highlighted by the experiments, orphan events with an associated noise have the potential to negatively affect the quality of our imputed prefixes, resulting in over- or underestimated non-conformance. Missing events can get masked by the orphan events, resulting in underestimated non-conformance. The magnitude of underestimation is somehow related to the number of missing events. Although the noise related to (consecutive) redundant events does not exist in the event logs of our experiments, with cognizance gained from the experiments, our approach is resilient toward such redundant events as it maps the orphan event to the transition bearing the shortest prefix. The potential of the out-of-order (or swapped) events as orphan events to negatively affect the quality of imputation depends on the orientation of the out-of-order event and usually causes non-conformance to be underestimated.

Building on the insights gained from the experiments, a relatively severe kind of noise affecting our prefix-imputation decisions would be *spurious* orphan events. An event is considered spurious if it seemingly does not fit the context of the rest of the trace (van Zelst et al., 2020). Highly out-of-order arrived event or an event belonging to an unreachable marking of the marking in which a case was forgotten is an example of this type of noise. For instance, consider a scenario where we forget a case *X* having the trace ⟨*A*⟩, with reference to the process model of Figure 2. Later, we observe an event (*X*, *K*) as an orphan event for case X. Our proposed prefix-imputation approach will impute the prefix ⟨**A, B, C, D, G, H, I, J**⟩ to the orphan event *K* for case *X*. Later, we observe the sequence of events ⟨*B*, *C*, *D*, *G*, *H*, *I*, *J*⟩ for case *X*, implying that the orphan event (*X*, *K*) was spurious. Any event observed after the orphan event will be marked as a log move, therefore causing a series of log moves and overestimated non-conformance. Interestingly, the prefix-alignments without prefix imputation in place will mark the spurious event as a log move, thereby correctly estimating the (non)conformance for trace *X*. The rationale behind our heuristic approach to deal with duplicate labels can be easily elaborated in this particular scenario. Suppose the spurious orphan event can be mapped to multiple transitions in the reference process model. Our heuristic approach will map the orphan event to the transition bearing the shortest prefix in comparison with the other candidate transitions. The imputed prefix being shortest in comparison with the other candidates will therefore cause less (wrongly) overestimated non-conformance.

It is pertinent to mention here that the negative effect of missing and out-of-order arrived orphan events is more pronounced in our experiments as the stream produced from static event logs does not get a continuous influx of new cases. The effect is expected to be far less in real streaming environments where new cases will keep arriving continuously and presumably best candidates for forgetting will always be available, resulting in a more favorable forgetting pattern.

## 6 Conclusion and Future Work

In this paper, we proposed a novel solution for bounding memory in event stream processing systems without falling prey to the missing-prefix problem. Our proposed approach selectively forgets reproducible cases in memory overflow situations and accordingly reconstructs the forgotten prefixes with the help of the normative reference process model on observing the orphan events. We evaluated the efficacy of our approach with synthetic and real event data. Despite some underestimation caused by noisy events, the results are reliable and reasonable in comparison with those of the approaches which lack a mechanism to deal with the missing-prefix problem.

We identified some areas for improvement and enhancement in our proposed approach. We envision an *imputation decision revision* mechanism as a remedial action for dealing with incorrect or partly correct imputation decisions resulting mainly from spurious orphan events, or label duplication. Till a certain event window after imputation, we shall observe if the imputed prefix and non-imputed parts of the trace are still in harmony, in light of the reference process model. If a disparity is observed, then the imputations shall be revisited.

Information about missing events cannot be established a priori as we lack knowledge about the past and future events of an orphan event. As a solution to avoid the masking of missing events, besides a process model, the statistical information of the events observed over a past case–based or time-based window can be stored. On the basis of this statistical information, an *adaptive* forgetting mechanism shall be able to recognize the potential problematic markings in the process model for which model moves are prevalent and subsequently cases in such markings shall not be forgotten.

In our proposed approach, we use the shortest model prefix for imputation. In some classes of processes, the shortest prefix may not always be *optimal*, and for such processes, the optimal prefix cannot be ascertained only through a process model. Priority-based processes, where different classes of customers are treated differently, are an example of such processes. For predicting optimal prefixes in such processes, in addition to a process model, we need to take contextual information into consideration. Therefore, we aim to incorporate all available information sources of the process into a machine learning predictive model to predict optimal prefixes for imputation of the orphan events. Additionally, the process stakeholders may be interested in high imputation precision in some critical parts of the process while being flexible in other non-critical areas of the process. A *personalized* forgetting mechanism, conceptualized by a domain expert, is therefore conceived as a relevant future work area.

## Data Availability

The synthetic event log of the CCC'19 process model is available on the link provided in the footnote^5^. The BPIC'12 and Road Traffic Fine Management real event data is publicly available on the 4TU research data repository (van Dongen, 2012; de Leoni and Mannhardt, 2015).

## Footnotes

1
https://github.com/rashidzaman84/MBprefiximputation.

2
www.processmining.org.

3
https://cpntools.org.

4
https://data.4tu.nl/articles/dataset/Conformance_Checking_Challenge_2019_CCC19_/12714932/1.
