# Supplementary material for: Prefix Imputation of Orphan Events in Event Stream Processing
Source: Front Big Data. 2021 Oct 6;4:705243. doi: 10.3389/fdata.2021.705243 (PMC8528154; doi:10.3389/fdata.2021.705243)
Supplement: Supplementary file 1 [file Presentation1.pdf]

# Supplementary Material

## 1 SUPPLEMENTARY FIGURES

The authors provide the figures of the process models used in the experimental evaluation on the following pages.

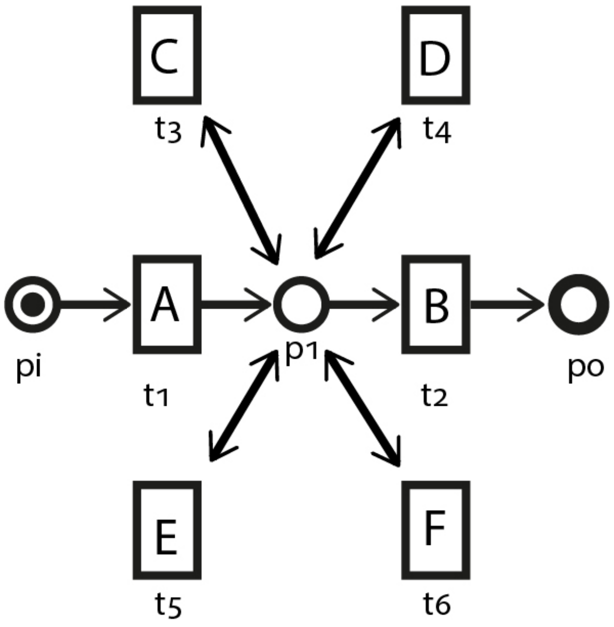


**Figure S1.** An example flower-like Petri net model

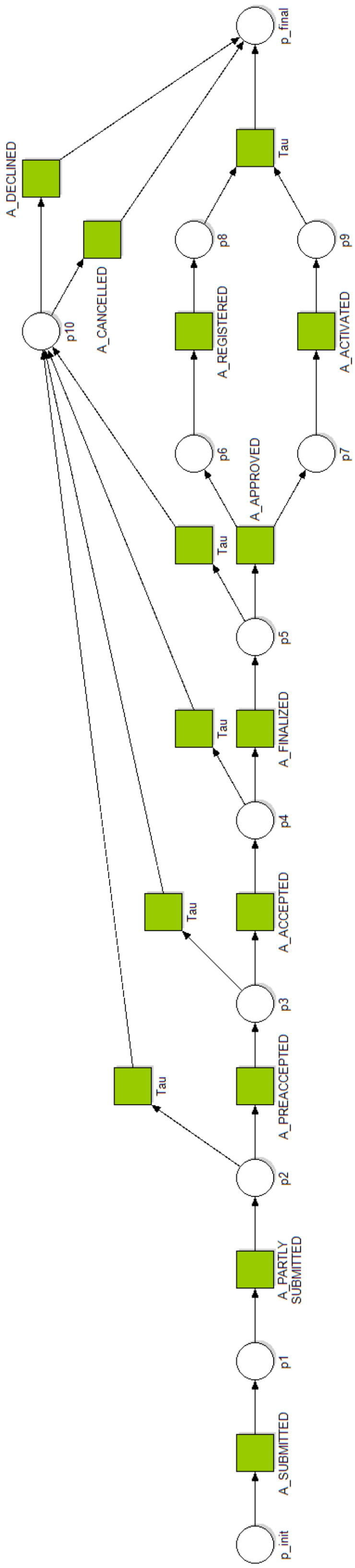


**Figure S2.** The process model of *Application* process of BPIC'12 event data

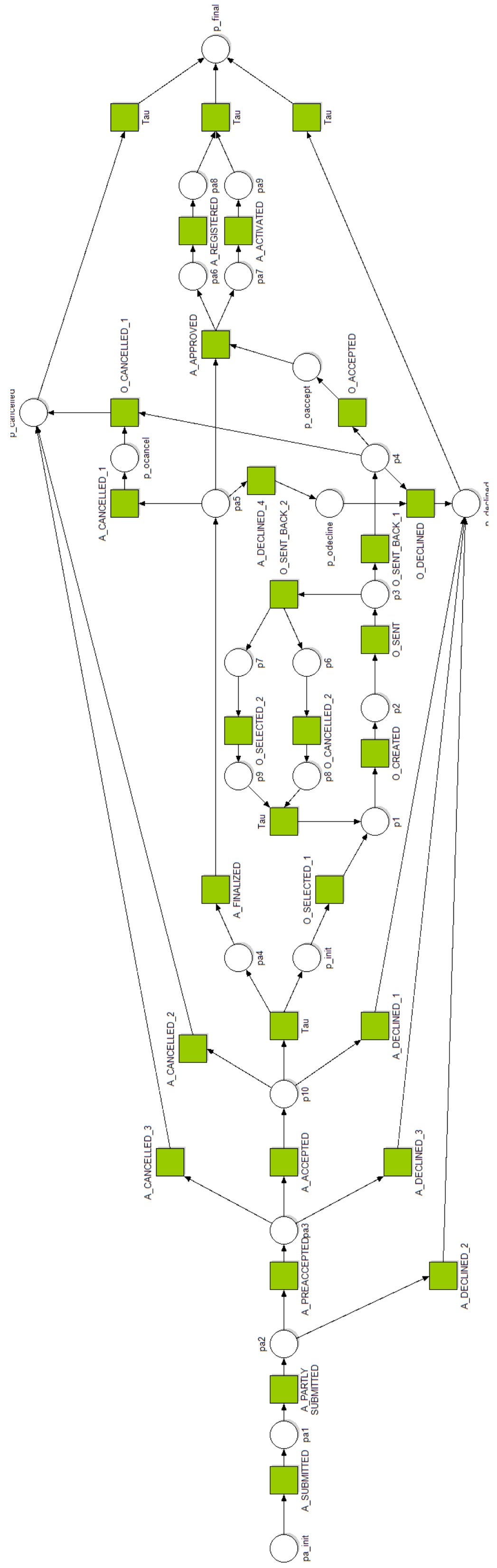


**Figure S3.** The process model of *Application* process and *Offer* sub-process of BPIC'12 event data

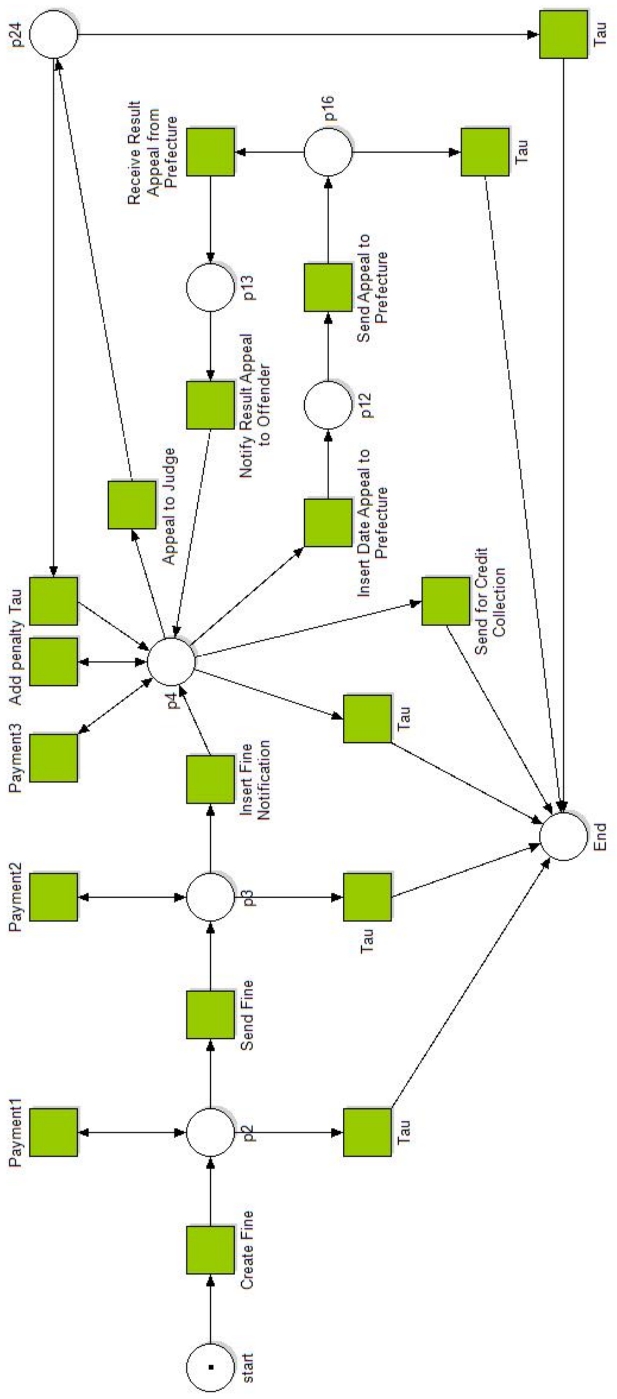


**Figure S4.** The process model of *Road Traffic Fine Management* process
